# Stress-induced inflammation evoked by immunogenic cell death is blunted by the IRE1α kinase inhibitor KIRA6 through HSP60 targeting

**DOI:** 10.1038/s41418-021-00853-5

**Published:** 2021-08-27

**Authors:** Nicole Rufo, Dimitris Korovesis, Sofie Van Eygen, Rita Derua, Abhishek D. Garg, Francesca Finotello, Monica Vara-Perez, Jan Rožanc, Michael Dewaele, Peter A. de Witte, Leonidas G. Alexopoulos, Sophie Janssens, Lasse Sinkkonen, Thomas Sauter, Steven H. L. Verhelst, Patrizia Agostinis

**Affiliations:** 1 Cell Death Research and Therapy Laboratory, Department of Cellular and Molecular Medicine, KU Leuven, Leuven, Belgium; 2 VIB Center for Cancer Biology Research, Leuven, Belgium; 3 Laboratory of Chemical Biology, Department of Cellular and Molecular Medicine, KU Leuven, Leuven, Belgium; 4 Laboratory of Protein Phosphorylation and Proteomics, Department of Cellular and Molecular Medicine and SyBioMa, KU Leuven, Leuven, Belgium; 5 Biocenter, Institute of Bioinformatics, Medical University of Innsbruck, Innsbruck, Austria; 6 Department of Life Sciences and Medicine, University of Luxembourg, Belvaux, Luxembourg; 7 ProtATonce Ltd, Science Park Demokritos, Athens, Greece; 8 Laboratory for Molecular Cancer Biology, Department of Oncology, KU Leuven, Leuven, Belgium; 9 Laboratory for Molecular Biodiscovery, Department of Pharmaceutical and Pharmacological Sciences, KU Leuven, Leuven, Belgium; 10 BioSys Lab, Department of Mechanical Engineering, National Technical University of Athens, Zografou, Greece; 11 Laboratory for ER stress and Inflammation, VIB Center for Inflammation Research and Department of Internal Medicine and Pediatrics, Ghent University, Ghent, Belgium; 12 AG Chemical Proteomics, Leibniz Institute for Analytical Sciences ISAS, e.V., Dortmund, Germany

**Keywords:** Chaperones, Target identification, Chemokines, Immune cell death, Signal transduction

## Abstract

Mounting evidence indicates that immunogenic therapies engaging the unfolded protein response (UPR) following endoplasmic reticulum (ER) stress favor proficient cancer cell-immune interactions, by stimulating the release of immunomodulatory/proinflammatory factors by stressed or dying cancer cells. UPR-driven transcription of proinflammatory cytokines/chemokines exert beneficial or detrimental effects on tumor growth and antitumor immunity, but the cell-autonomous machinery governing the cancer cell inflammatory output in response to immunogenic therapies remains poorly defined. Here, we profiled the transcriptome of cancer cells responding to immunogenic or weakly immunogenic treatments. Bioinformatics-driven pathway analysis indicated that immunogenic treatments instigated a NF-κB/AP-1-inflammatory stress response, which dissociated from both cell death and UPR. This stress-induced inflammation was specifically abolished by the IRE1α-kinase inhibitor KIRA6. Supernatants from immunogenic chemotherapy and KIRA6 co-treated cancer cells were deprived of proinflammatory/chemoattractant factors and failed to mobilize neutrophils and induce dendritic cell maturation. Furthermore, KIRA6 significantly reduced the in vivo vaccination potential of dying cancer cells responding to immunogenic chemotherapy. Mechanistically, we found that the anti-inflammatory effect of KIRA6 was still effective in IRE1α-deficient cells, indicating a hitherto unknown off-target effector of this IRE1α-kinase inhibitor. Generation of a KIRA6-clickable photoaffinity probe, mass spectrometry, and co-immunoprecipitation analysis identified cytosolic HSP60 as a KIRA6 off-target in the IKK-driven NF-κB pathway. In sum, our study unravels that HSP60 is a KIRA6-inhibitable upstream regulator of the NF-κB/AP-1-inflammatory stress responses evoked by immunogenic treatments. It also urges caution when interpreting the anti-inflammatory action of IRE1α chemical inhibitors.

## Introduction

In response to anticancer treatments, stressed or dying cancer cells engage in a complex dialogue with the tumor microenvironment, which can ultimately stimulate or suppress inflammatory and immune responses. A subset of anticancer therapies (including anthracyclines and photodynamic therapy) [1] stimulates cancer cell-autonomous pathways leading to a form of immunostimulatory apoptosis, called immunogenic cell death (ICD). A hallmark of ICD is the concomitant stimulation of reactive oxygen species (ROS) and endoplasmic reticulum (ER) stress, which together evoke the surface exposure or release of damage-associated molecular patterns (DAMPs) [1, 2]. DAMPs act as danger signals, which are sensed and decoded by dendritic cells (DCs) through the binding to specific pathogen recognition receptors (PRRs) on their surface [2], ultimately triggering an antigen-specific antitumor immunity.

Scrutiny of the mechanistic underpinnings of ICD and in vivo studies have disclosed that perturbation of ER homeostasis triggering the PERK/eIF2α-P axis of the unfolded protein response (UPR), orchestrates danger signaling [3, 4]. In contrast, the role of the IRE1α during ICD remains insufficiently understood.

However, while the critical role of danger signals has been validated in preclinical and in some clinical settings [5], much less information is available on the molecular pathways controlling the proinflammatory outputs of the UPR elicited by immunogenic treatments. Moreover, whether a transcriptional inflammatory signature portrays a distinguished trait of immunogenic therapies is still unexplored.

Growing evidence indicate that inflammation evoked by perturbations of ER homeostasis ignited by immunogenic therapies may play a distinct role from danger signals [6]. In response to immunogenic chemotherapies, CCL2 release by the stressed cancer cells boosts the initial phase of the immunogenic response by enabling the recruitment of antigen-presenting cells [7]. However, CCL2 also evokes secondary inflammation, which ultimately drives angiogenesis, metastasis, and recurrence [8, 9]. Cancer vaccines generated by exposing cancer cells to ICD favor the recruitment of neutrophils at the site of vaccination, through the release of a set of chemokines patterning cellular responses to pathogens [10]. However, in a tumor context, chemokines driving neutrophil infiltration into the tumor may favor tumor immunoescape by shielding cancer cells from cytotoxic T cells [11]. Hence, sterile inflammatory responses elicited by stressed cancer cells may exert context-dependent effects on the tumor microenvironment and the overall output of immunogenic therapies.

This creates an urgent need to delineate cancer cell-autonomous molecular pathways responsible for the expression of these inflammatory factors.

## Results

### Immunogenic treatments are hallmarked by a proinflammatory transcriptional signature

We set out to investigate the transcriptional profile of cancer cells responding to mitoxantrone (MTX) or hypericin-based photodynamic therapy (Hyp-PDT), as prototypes of immunogenic treatments [12], whereas cisplatin (CDDP) served as paradigm of poorly ICD inducer [12]. These treatments induced similar kinetics of apoptosis in the human melanoma A375 cell line (Supplementary Fig. 1A) but only MTX and Hyp-PDT elicited classical in vitro markers of immunogenic apoptosis, including surface-exposed CRT (Supplementary Fig. 1B), ATP release—particularly after Hyp-PDT (Supplementary Fig. 1C)—and cytoplasmic redistribution of nuclear HMGB1 prior to its passive release (Supplementary Fig. 1D, E), confirming previous studies [3, 13–15]. Co-incubation of dying melanoma cells with human monocyte-derived DCs indicated that only MTX and Hyp-PDT increased surface expression of the DC-maturation markers CD86 and HLA-DR (Supplementary Fig. 1F).

**Fig. 1:**
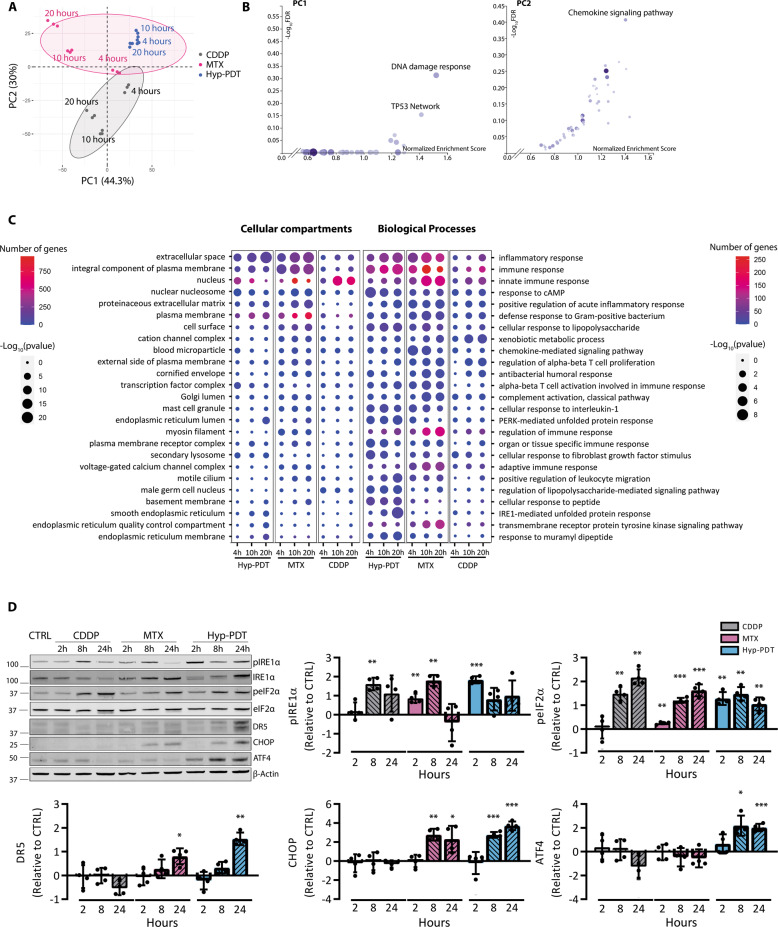
Immunogenic treatments are hallmarked by a proinflammatory transcriptional program. **A** Principal component analysis (PCA) performed on the rlog-normalized RNA-seq counts from A375 cells at 4, 10, and 20 h after treatment with CDDP, MTX, or Hyp-PDT. Concentrations of the drugs are reported in the materials and methods section and remain constant through all the experiments. Three independent biological replicates are represented for each timepoint and treatment. Ellipses draw 90% confidence area for immunogenic (pink) and nonimmunogenic (CDDP, gray) treatments. **B** Geneset enrichment analysis (GSEA) of Wikipathways Cancer showing the −log_10_(FDR) and Normalized enrichment scores of the genesets positively associated to input gene lists. **C** Results of the Gene ontology (GO) analysis based on genes significantly upregulated in the RNA-seq dataset in samples treated with CDDP, MTX, or Hyp-PDT compared to time-matched untreated control. Top 25 most variable GO terms relative to biological process and cellular compartment are represented. Dot sizes and colors represent −log_10_(*p* value) of the enrichment of each term in the different samples and number of genes, respectively. **D** Representative time course western blot and relative quantifications for markers underlining UPR induction after treatment with CDDP, MTX, or Hyp-PDT. β-actin was used as normalization parameter; peIF2α is normalized over total levels of eIF2α. Values represent mean ± SD of the log_2_(fold change) over control of *n* = 4 independent biological replicates. Data are analyzed by one-sample *t*-test against hypothetical control value set to 0. *p < 0.05, **p < 0.01, ***p < 0.001.

We then interrogated the time-dependent changes in the transcriptome of the treated A375 cells by bulk-RNA sequencing (RNA-seq) analysis at 0 h (untreated), 4 h (preapoptotic), 10 h (early apoptotic), and 20 h (late apoptotic) post-treatment (Supplementary Fig. 1G). Bidimensional principal component analysis (PCA) indicated that MTX and Hyp-PDT treatments clearly separate on the second dimension (PC2) from CDDP, which accounted for 30% of the total variance (Fig. 1A). Gene Set Enrichment Analysis (GSEA) [16] using the WikiPathway Cancer geneset and weighing the genes for their contribution to PC1 and PC2, showed that PC1, clustering the two genotoxic agents CDDP/MTX, was enriched in the “DNA-damage response” geneset (Fig. 1B). Instead, “chemokine signaling pathway” emerged as predominantly enriched gene signature from PC2, which clustered MTX and Hyp-PDT (Fig. 1B). Gene Ontology (GO) analysis performed on the totality of significantly upregulated genes pinpointed “inflammatory response/immune responses” and “extracellular space” as the most significant GO terms almost exclusively associated with immunogenic treatments throughout the time course (Fig. 1C). PERK- and IRE1α-mediated UPR responses were particularly sustained after Hyp-PDT (Fig. 1C, D), a primarily ER-targeted ROS-generating treatment [3], which elicited markers of terminal UPR, including ATF4, CHOP, and its downstream target DR5 (TRAIL-R2) (Fig. 1D). MTX stimulated the early phosphorylation of IRE1α (Fig. 1D) and eIF2α, a signature of the integrated stress response (ISR) [17], and expression of DR5 and CHOP at later timepoints (Fig. 1D). In contrast, CDDP failed to induce terminal UPR markers even if it elevated at later timepoints the phosphorylation of IRE1α and eIF2α (Fig. 1D), likely as a secondary effect of cytosolic generation of ROS [18].

Together these results suggest that immunogenic treatments engaging the UPR/ISR evoke a distinct transcriptional signature hallmarked by the expression of a subset of proinflammatory transcripts.

### Transcriptional upregulation of a subset of chemokines is a hallmark of immunogenic treatments

**Fig. 2:**
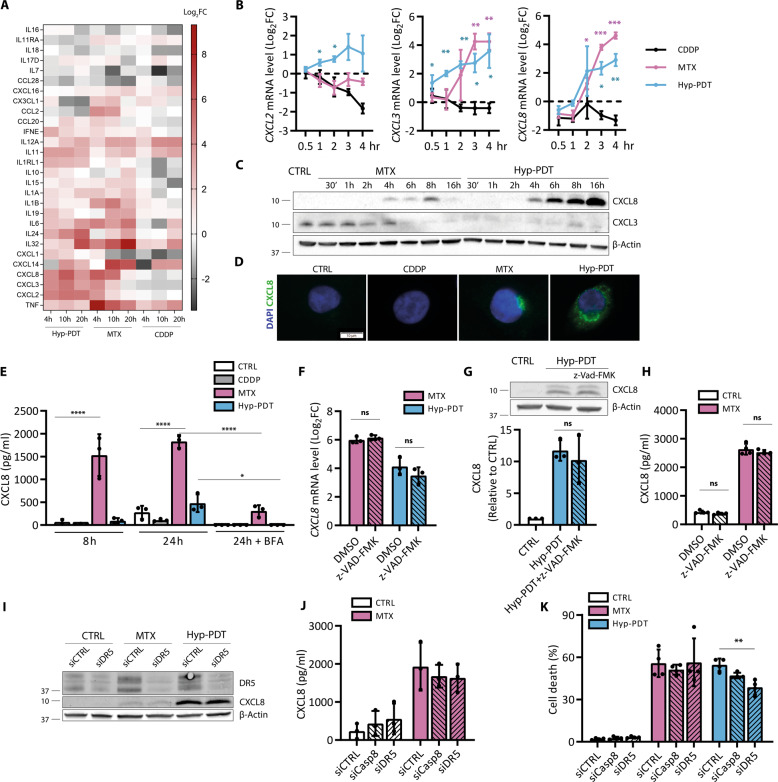
Immunogenic treatments elicit chemokine production independent of cell death. **A** Heatmap obtained from RNA-seq data representing the log_2_(fold changes) of cytokine and chemokines genes upon treatment with CDDP, MTX, or Hyp-PDT compared to time-matched untreated controls. **B** Chemokine transcription was evaluated at short timepoints after treatment with CDDP, MTX, or Hyp-PDT with a time course analysis performed by qPCR. Data are expressed as log_2_(fold change) compared to time-matched untreated control. Asterisks are color-matched to the treatment. **C** Intracellular CXCL8 accumulation and CXCL3 depletion at the indicated timepoints upon treatment with MTX or Hyp-PDT. **D** Intracellular accumulation of CXCL8 (green) in control (CTRL) condition or 8 h after treatment with CDDP, MTX, or Hyp-PDT. Nuclei were counterstained with DAPI (blue). Scale bar: 10 μm. **E** CXCL8 secretion was measured by ELISA in conditioned medium at 8 h and 24 h after treatment with CDDP, MTX, or Hyp-PDT. CXCL8 secretion at 24 h after treatment was blocked by Brefeldin A (BFA, 50 ng/ml). **F–H** Evaluation of the impact of z-VAD-FMK (50 μM) on CXCL8 transcription (**F**), intracellular protein accumulation (**G**) and secretion (**H**) after treatment with Hyp-PDT or MTX was assessed by qPCR, western blot, and ELISA, respectively. Values are represented as log_2_(fold change) in (**F**) and fold change over untreated control in (**G**). **I** Impact of siRNA mediated DR5 silencing (siDR5) on CXCL8 protein production measured by western blot 24 h after treatment with MTX or Hyp-PDT compared to scrambled siRNA (siCTRL). **J** Secretion of CXCL8 in conditioned medium of siDR5, siCasp8, and siCTRL A375p cells measured by ELISA 24 h after treatment with MTX. **K** Cell death was assessed in siDR5, siCasp8, and siCTRL A375p cells 24 h after treatment with MTX or Hyp-PDT. In all western blots β-actin was used as loading control. In all graphs values are presented as mean ± SD of at least *n* = 3 independent biological replicates. Data are analyzed by one-sample *t*-test in (**B**), One-way ANOVA followed by Dunnett’s multiple comparison test in (**E, K**) and two-tailed Student’s *t*-test in (**F–H**), *p < 0.05,**p < 0.01, ***p < 0.001, ****p < 0.0001, ns not significant.

To further unravel a distinguished ICD-associated transcriptional signature, we focused on *CXCL8*, *CXCL3*, and *CXCL2*, proinflammatory chemokines with relevant autocrine and paracrine roles [19]. We validated that these chemokines were differentially co-upregulated in response to ICD, while simultaneously co-repressed by CDDP (Fig. 2A, B). We then focused on the production of CXCL3 and CXCL8 as these chemokines were most significantly induced by both MTX and Hyp-PDT. Intracellular baseline levels of CXCL3 significantly decreased in response to both treatments (Fig. 2C, Supplementary Fig. 2A), and were partially recovered by inhibition of the proteasome (Supplementary Fig. 2A), suggesting its fast degradation upon induction. CXCL8 levels instead increased rapidly after treatments (Fig. 2C, D). After MTX the intracellular accumulation of CXCL8 was accompanied by its rapid secretion (Fig. 2E), which occurred via canonical anterograde transport (Fig. 2E, Supplementary Fig. 2B). These data suggest that CXCL8 expression is predominantly an early response to MTX-induced stress, as its intracellular levels (Fig. 2C) and secretion (Fig. 2E) dropped at late time points. In line with the reduced secretory ability of PDT-stressed cells [3, 20], CXCL8 accumulated predominantly intracellularly (Fig. 2C, E).

Blocking apoptosis by the pan-caspase inhibitor z-VAD-FMK (Supplementary Fig. 1A) did not affect CXCL8 expression at the RNA (Fig. 2F) or protein levels (Fig. 2G) or its release after MTX (Fig. 2H). Similar results were obtained in MTX-treated HeLa cells (Supplementary Fig. 2C). Thus increasing the fraction of surviving cells does not enhance CXCL8 production by the stressed cells.

PERK-mediated and CHOP-induced upregulation of DR5 (TRAIL-R2) following canonical ER stress-inducing agents and taxol, has been shown to trigger cytokine production through a DR5/caspase-8/FADDosome-driven pathway [6, 21]. Congruently with the differential kinetics of CXCL8 and DR5 upregulation (e.g., 2 and 24 h, respectively, Figs. 2B, 1D), siRNA-mediated silencing of DR5 (about 80%, Fig. 2I, Supplementary Fig. 2D) or caspase-8 (about 75%, Supplementary Fig. 2C), removing both its activity and scaffolding function, did not affect CXCL8 production after Hyp-PDT or MTX (Fig. 2I, J, Supplementary Fig. 2D). Downregulation of DR5 expression (Fig. 2K) or pharmacological blockade of PERK (Supplementary Fig. 2E) reduced to some extent cell death after Hyp-PDT, consistent with the proapoptotic role of this UPR axis after ROS-induced ER stress [22], while caspase-8 silencing did not impact overall cell death (Fig. 2K).

Collectively, these results argue that CXCL8 production following immunogenic treatments occurs as a result of a DR5- and caspase-independent, premortem stress response.

### CXCL8 induction following immunogenic treatments requires NF-κB and cJUN

**Fig. 3:**
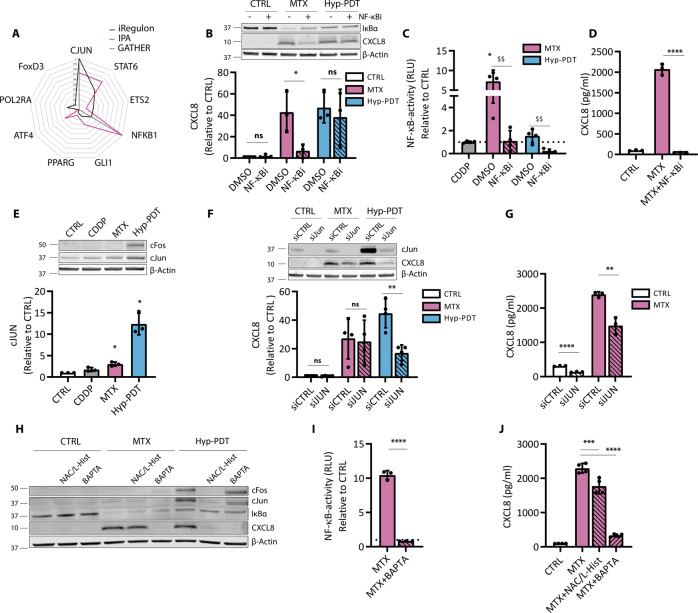
CXCL8 production is mediated by cJUN and NF-κB. **A** In silico prediction of transcription factors involved in the regulation of genes belonging to the “extracellular space” GO term significantly upregulated by both MTX and Hyp-PDT. The transcription factors reported are derived from iRegulon prediction, and independent scores of the same transcription factors by IPA and Gather are provided. Data are reported after min–max normalization. **B** Impact of BAY11-7082 (10 μM) on degradation of IκBα and CXCL8 intracellular protein accumulation 4 h after treatment with MTX or Hyp-PDT. **C** NF-κB activity measured by luciferase assay in A375 cells stably expressing the reporter 4 h after treatment with CDDP, MTX, or Hyp-PDT in the presence or absence of BAY11-7082 (10 μM). Data are expressed as fold change compared to untreated control, indicated with a dotted line. **D** CXCL8 secretion measured by ELISA in conditioned medium from A375 cells with or without co-incubation with BAY11-7082 (10 μM) 24 h after treatment with MTX. **E** Intracellular levels of cFOS and cJUN 4 h after treatment with CDDP, MTX, or Hyp-PDT. Data are expressed as fold change over untreated control. **F** Impact of siRNA mediated knockdown of cJUN (siJUN) with respect to scramble siRNA (siCTRL) on intracellular CXCL8 accumulation 4 h after treatment with MTX or Hyp-PDT. Data are expressed as fold change over control incubated with siCTRL. **G** CXCL8 secretion was measured by ELISA in the conditioned medium from A375 cells with siRNA-mediated cJUN knockdown 24 h after treatment with MTX. **H** Impact of ROS inhibitors N-Acetyl-L-Cysteine (NAC, 5 mM))/L-Histidine (25 mM) and cell permeable calcium chelator BAPTA-AM (10 μM) on the upregulation of cJUN and cFOS, degradation of IκBα and CXCL8 production 4 h after treatment with MTX or Hyp-PDT. **I** NF-κB activity measured by luciferase assay in A375 cells stably expressing the reporter 4 h after treatment with MTX in presence or absence of BAPTA-AM (10 μM). Data are expressed as fold change compared to untreated control, indicated with a dotted line. **J** CXCL8 secretion measured by ELISA in conditioned medium from A375 cells with or without co-incubation with NAC (5 mM)/L-Histidine (25 mM) or BAPTA-AM (10 μM) 24 h after treatment with MTX. In all western blots β-actin was used as loading control. In all graphs values are presented as mean ± SD of at least *n* = 3 independent biological replicates. Data are analyzed by one-sample *t*-test in (**C, E**), two-tailed Student’s *t*-test in (**B, C, D, F, G, I**), and one-way ANOVA followed by Dunnett’s multiple comparison test in (**J**). *p < 0.05, **p < 0.01, ***p < 0.001, ****p < 0.0001, ^$$^*p* < 0.001 versus inhibitor-free treatment, ns not significant.

To predict putative transcription factors (TFs) regulating the common proinflammatory trait of immunogenic treatments, we queried three different bioinformatics tools (IPA, iRegulon and GATHER). Only two TFs were conmmonly present in the top 10, namely the AP-1 member cJUN and NF-κB (Fig. 3A).

Consistently, MTX caused degradation of IκBα (Fig. 3B) and NF-κB activation, as measured by a luciferase reporter assay (Fig. 3C). MTX-stimulated CXCL8 production (Fig. 3B, D) and NF-κB activation (Fig. 3C) were blunted by the IKK inhibitor BAY11-7082, which blocked IκBα degradation (Fig. 3B) but had otherwise no effect in unstimulated conditions (not shown). Hyp-PDT failed to stimulate NF-κB signaling (Fig. 3B, C), and accordingly CXCL8 induction was unaffected by NF-κB inhibition (Supplementary Fig. 1A). Instead, Hyp-PDT induced a robust upregulation of the main AP-1 members cJUN and cFOS, while MTX partially induced cJUN expression (Fig. 3E). Silencing cJUN decreased intracellular expression of CXCL8 in response to Hyp-PDT (Fig. 3F) and reduced CXCL8 secretion after MTX (Fig. 3G). The complete inhibition of CXCL8 production by the blockade of NF-κB (Fig. 3C) suggests that following MTX, NF-κB operates as a dominant proinflammatory TF [23]. Accordingly, NF-κB inhibition significantly decreased cJUN upregulation in MTX-treated cells (Supplementary Fig. 3B). In all cases CDDP failed to stimulate these TFs (Fig. 3C, E).

Interestingly, impairing intracellular Ca^2+^ levels or ROS generation, two apical signals elicited by immunogenic treatments [3, 24], curtailed both NF-κB and AP-1 mediated CXCL8 production following MTX or Hyp-PDT, respectively (Fig. 3H-J).

Together these results show that the proinflammatory output elicited by immunogenic treatments is mediated by NF-κB and AP-1 signaling.

### Pharmacological inhibition of IRE1α kinase activity curtails ICD-induced proinflammatory responses

Both PERK and IRE1α pathways mediate NF-κB and AP-1 driven proinflammatory cytokine production [25] and pharmacological inhibitors of these ER stress sensors have been used in vivo to ameliorate the pathological output of the UPR [26].

**Fig. 4:**
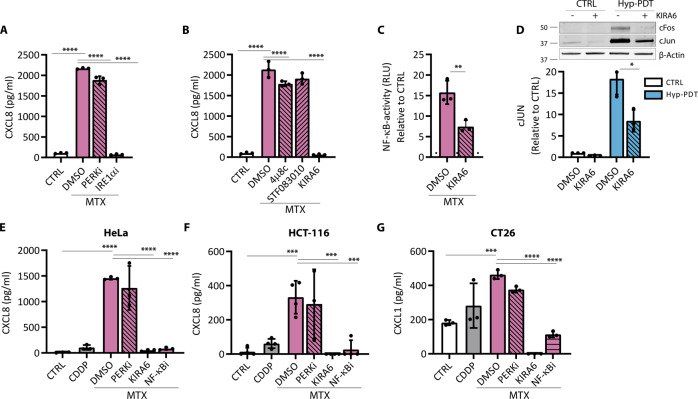
The IRE1α kinase inhibitor KIRA6 blunts CXCL8 production after immunogenic treatment. **A** CXCL8 secretion was measured by ELISA in conditioned medium of A375 cells 24 h after treatment with MTX in co-incubation with the PERK inhibitor GSK2606414 (1 μM) or the IRE1α kinase inhibitor KIRA6 (1 μM). **B** CXCL8 secretion was measured by ELISA in conditioned medium of A375 cells 24 h after treatment with MTX in co-incubation with the IRE1α RNAse inhibitors 4μ8C (100 μM) and STF-083010 (50 μM) or IRE1α kinase inhibitor KIRA6 (1 μM). **C** NF-κB activity measured by luciferase assay in A375 cells stably expressing the reporter 4 h after treatment with MTX in the presence or absence of KIRA6 (1 μM). Data are expressed as fold change compared to untreated control, indicated with a dotted line. **D** Impact of KIRA6 (1 μM) on intracellular levels of cJUN and cFOS in basal condition and 4 h after treatment with Hyp-PDT. β-actin was used as loading control. **E**, **F** CXCL8 secretion was measured by ELISA in conditioned medium of HeLa and HCT-116 cells 24 h after treatment with MTX in co-incubation with GSK2606414 (1 μM), KIRA6 (1 μM) or BAY11-7082 (10 μM). **G** CXCL1 secretion was measured by ELISA in conditioned medium of murine CT26 cells 24 h after treatment with MTX in co-incubation with GSK2606414 (1 μM), KIRA6 (1 μM), and BAY11-7082 (10 μM). In all graphs values are presented as mean ± SD of at least *n* = 3 independent biological replicates. Data are analyzed by one-way ANOVA followed by Dunnett’s multiple comparison test in all the graphs except for two-tailed Student’s *t*-test in (**C, D**). *p < 0.05, **p < 0.01, ***p < 0.001, ****p < 0.0001.

PERK inhibition only marginally affected CXCL8 production by MTX (Fig. 4A) or had no effect after Hyp-PDT (Supplementary Fig. 4A). Blocking the autophosphorylation of IRE1α by KIRA6, curtailed CXCL8 production in response to both MTX and Hyp-PDT (Fig. 4A, Supplementary Fig. 4A).

The imidazopyrazine KIRA6 by interacting with the ATP-binding site of IRE1α inhibits its autophosphorylation (Supplementary Fig. 4B) and oligomerization, and consequently the RNase activity of IRE1α [27] (Supplementary Fig. 4C). However, blocking IRE1α RNase activity by the chemical inhibitors 4μ8C or STF-083010 (Supplementary Fig. 4C) had no or only marginal effects on the production of CXLC8 following MTX (Fig. 4B), which does not induce XBP1 cleavage (Supplementary Fig. 4C, D), or Hyp-PDT (Supplementary Fig. 4E).

These results suggested that the IRE1α kinase activity drives inflammation, possibly through its reported ability to recruit scaffolding proteins independent of its RNAse activity [28, 29].

KIRA6 significantly reduced both NF-κB activity following MTX (Fig. 4C) and AP-1 induction following Hyp-PDT (Fig. 4D). We then confirmed these findings in other cell lines (HeLa, HCT-116 and murine CT26), where only the blockade of NF-κB by BAY11-7082 and of the IRE1α kinase by KIRA6 abolished MTX-induced CXCL8 and CXCL1 (a murine ortholog of CXCL8) secretion (Fig. 4E-G).

Assessment of a wider panel of cytokines released by the A375 cells in response to MTX by multiplexed ELISA showed the ability of KIRA6 to potently inhibit NF-κB-induced proinflammatory targets, but not of an interferon-inducible CXCL10 [30] (Supplementary Fig. 4F).

### KIRA6 suppresses the immunogenic potential of cancer cells responding to immunogenic chemotherapy

**Fig. 5:**
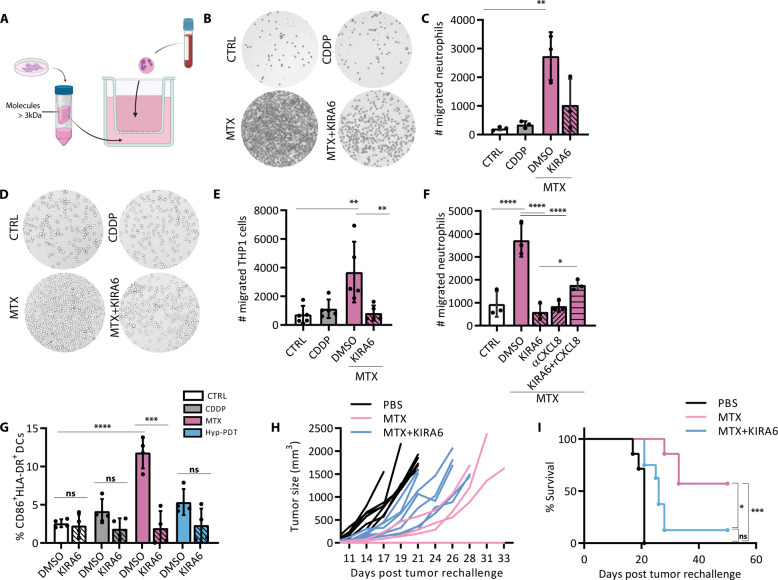
KIRA6 affects the immunogenicity of chemotherapy. **A** Scheme of the transwell migration experimental setup. **B, C** Transwell migration assay of human neutrophils exposed for 2 h to conditioned medium of A375 cells treated for 24 h with MTX in the presence or absence of KIRA6 (1 μM). **D, E** Representative pictures and relative quantification of the transwell migration assay of human macrophage-like THP1 cell line exposed for 4 h to conditioned medium of A375 cells treated for 24 h with MTX in the presence or absence of KIRA6 (1 μM). **F** Neutrophil migration was assessed after neutralization of CXCL8 secreted upon treatment with MTX with a αCXCL8-neutralizing antibody (0.5 μg/ml) or after addition of recombinant CXCL8 (10 ng/ml) in MTX + KIRA6 conditioned medium. **G** Dendritic cell maturation was assessed by measuring the surface expression of CD86 and HLA-DR by FACS 24 h after co-incubation with the ‘cell free’ medium of A375 cells treated for 24 h with CDDP, MTX, or Hyp-PDT in the presence or absence of KIRA6 (1 μM). **H, I** BALB/c mice immunized with PBS or with CT26 treated for 24 h with MTX in the presence or absence of KIRA6 (1 μM) were rechallenged with live CT26 tumor cells, and tumor growth (**H**) and survival (**I**) were monitored. *N* = 7-8mice/condition. Mice that did not develop tumor (*n* = 4 out 7 in MTX group and *n* = 1 out of 8 in MTX + KIRA6 group) are shown as a flat line on the *x*-axis in (**H**). In all graphs values are presented as mean ± SD of at least *n* = 3 independent biological replicates. Data are analyzed by one-way ANOVA followed by Dunnett’s multiple comparison test in all the graphs except for two-tailed Student’s *t*-test in (**G**) to compare the effect of the inhibitor on the treatment, and Log-rank Mantel-Cox in (**I**). *p < 0.05, **p < 0.01, ***p < 0.001, ****p < 0.0001, ns not significant.

CXCL8 potently orchestrates neutrophil migration and activation through its interaction with CXCR1 and CXCR2 class of G-protein-coupled receptors [19]. Since Hyp-PDT treated cells had impaired CXLC8 secretion (Fig. 2E), we evaluated the effects of KIRA6 on the ability of the supernatants of MTX-treated A375 cells to recruit human neutrophils and monocytes. Conditioned medium from CDDP-treated cells provided a negative control. To exclude direct effects of the drug and chemical inhibitor on neutrophils, we processed the conditioned media to extensive rounds of sieving to retain only components heavier than 3 kDa, thus eliminating all small molecules and drugs while preserving chemokines (typically in the range of 7–15 kDa) (Fig. 5A). We refer to this fraction as ‘cell-free’ medium. Congruently with the results shown in Figs. 2–4, the ‘cell-free’ medium from MTX-treated cancer cells elicited robust recruitment of neutrophils (Fig. 5B, C), whereas that from CDDP-treated cells failed to do so. Treatment of cancer cells with MTX in the presence of KIRA6 abolished the chemoattractant ability of their conditioned media (Fig. 5B, C). Similar results were obtained by assessing the transmigration capacity of the human monocyte-like cell line THP1 (Fig. 5D, E). Antibody-based neutralization of CXCL8 blocked neutrophils chemotaxis elicited by the conditioned media of MTX-treated cancer cells, to the same extent as that of the untreated cells or MTX-treated cells in the presence of KIRA6 (Fig. 5F). Exogenous addition of recombinant CXCL8 to the MTX-KIRA6 ‘cell-free’ medium partially recovered neutrophil transmigration ability (Fig. 5F), suggesting that recombinant CXCL8 may not fully mimic the native conformation of the secreted CXCL8 [31].

Furthermore, only the ‘cell-free’ medium from MTX-treated A375 cells caused a significant upregulation of the maturation markers CD86 and HLA-DR on the surface of DCs (Fig. 5G), and pretreatment with KIRA6 abolished the ability of MTX-derived conditioned medium to induce DC maturation (Fig. 5G).

We then tested the impact of KIRA6 on the overall immunogenic potential of MTX-treated murine colon carcinoma CT26 cells using a prophylactic vaccination setup in syngeneic mice. In CT26 cells KIRA6 enhanced MTX-induced cell death (Supplementary Fig. 5A) with a parallel strengthening of both ATP release and CRT surface exposure (Supplementary Fig. 5B, C). The release of HMGB1 and other immunomodulatory factors, such as the chaperones HSP60, HSP70, HSP90, instead was overall unaffected by KIRA6 (Supplementary Fig. 5D). To assess the antitumor vaccination potential of dying cancer cells exposed to MTX or MTX + KIRA6 (KIRA6 in the absence of MTX, did not induce cell death) we then injected CT26 stressed/dying cells subcutaneously in the flank of immunocompetent mice. Nonvaccinated, CTRL mice received only PBS.

Mice immunized with MTX-treated CT26 cells showed reduced tumor growth following rechallenging of live CT26 cells as opposed to nonimmunized mice (Fig. 5H, I), indicating the induction of an efficient anticancer vaccination response. In contrast, mice immunized with MTX + KIRA6-treated CT26 cells displayed a severe drop in their tumor-rejecting capability (from 60 to 12%) (Fig. 5H, I).

Together these results indicate that KIRA6 mediated inhibition of the proinflammatory trait elicited by MTX has profound repercussion on immune cell recruitment, DC activation, and the vaccination potential of immunogenic chemotherapy.

### The anti-inflammatory effects of KIRA6 are independent of IRE_1_

**Fig. 6:**
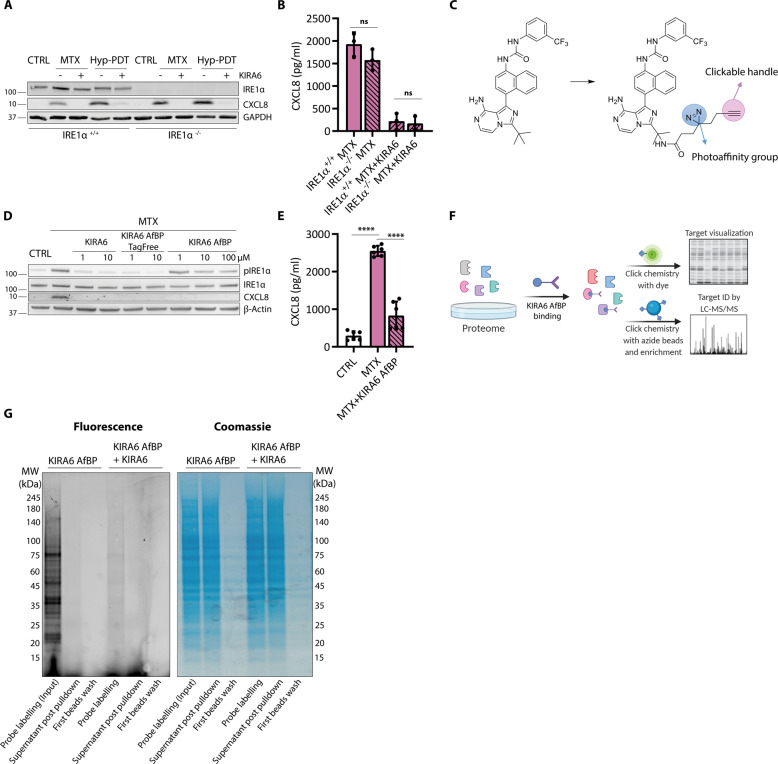
KIRA6 modulates CXCL8 production in an IRE1α-independent pathway. **A** KIRA6 (1 μM) mediated blocking of CXCL8 production and intracellular accumulation 4 h after treatment with MTX or Hyp-PDT in both IRE1α^−/−^ or IRE1α^+/+^ A375 cells. **B** CXCL8 secretion was measured by ELISA 24 h after treatment with MTX in conditioned medium of IRE1α^−/−^ or IRE1α^+/+^ A375 cells with or without incubation with KIRA6 (1 μM). **C** Molecular structure of the modified KIRA6 (KIRA6 Affinity-based Probe, KIRA6 AfBP) used for off-target protein identification. **D** Comparison of the ability of KIRA6, KIRA6 AfBP and the intermediate tag-free KIRA6 AfBP (without photoaffinity group and clickable handle) in inhibiting CXCL8 production and IRE1α phosphorylation 4 h after treatment with MTX in A375 cells at the indicated concentrations. **E** CXCL8 secretion was measured by ELISA in conditioned medium of A375 cells 24 h after treatment with MTX with or without co-incubation with KIRA6 AfBP (10 μM). **F** Scheme of the experimental workflow used to identify the KIRA6 off-targets by using the modified KIRA6 AfBP. **G** Representative gel assessing the efficacy of protein off-target identification workflow in protein lysates of A375 cells. Lane 1 shows multiple fluorescent bands indicating KIRA6 AfBP (10 μM) labelled proteins. Lane 2 shows selective depletion of KIRA6 AfBP labeled proteins following azide beads pull-down indicated by decrease of fluorescent signal but a comparable amount of proteins (Coomassie). Lane 3 shows efficient removal of the most abundant aspecific proteins (Coomassie) but absence of removal of labeled proteins (fluorescence). Lane 4 shows that KIRA6 AfBP protein binding is outcompeted by co-incubation with KIRA6 (10 μM) suggesting overlapping targets. (Table 1) Top 15 off-target candidates obtained by LC-MS/MS scored by total spectral counts. In the graphs values are presented as mean ± SD of at least *n* = 3 independent biological replicates and analyzed by two-tailed Student’s *t*-test,****p < 0.0001, ns not significant.

To validate the effects of KIRA6 we next generated IRE1α knockout (IRE1^−/−^) A375 cells through CRISPR/Cas9 gene editing. Efficient IRE1α knockout was assessed by lack of detectable IRE1α protein and absent XBP1s cleavage (Fig. 6A, Supplementary Fig. 6A). To our surprise, IRE1α^−/−^ A375 cells were as proficient as their IRE1α^+/+^ counterparts in promoting CXCL8 production following treatment with either MTX or Hyp-PDT (Fig. 6A, B). Moreover, KIRA6 was still able to block CXCL8 levels in IRE1α^−/−^ A375 cells exposed to MTX or Hyp-PDT (Fig. 6A, B). Similar results were obtained when IRE1α was downregulated by siRNA treatment in A375 cells (Supplementary Fig. 6B), in IRE1α^−/−^ MEFs (Supplementary Fig. 6C), or in CT26 cells with stable shRNA-mediated IRE1α downregulation (Supplementary Fig. 6D). Together these results indicated a hitherto unknown off-target effector of the anti-inflammatory activity of KIRA6 following immunogenic treatments.

To identify the off-target effector of KIRA6, we utilized a novel photoaffinity and clickable KIRA6 probe (KIRA6 AfBP) (Fig. 6C), which we recently synthesized [32].

We first confirmed that KIRA6 AfBP suppressed CXCL8 levels from MTX-treated cells to an extent similar to KIRA6 (Fig. 6D, E). Interestingly, the inclusion of the tag (i.e., photoreactive group + clickable handle) prevented the binding to IRE1α in live cells, since phosphorylation of IRE1α was still detectable when using KIRA6 AfBP, whereas it was suppressed by the tag-free KIRA6 AfBP precursor (Fig. 6D). This suggests that in A375 cells, KIRA AfBP is only able to bind to the off-target partner(s) of KIRA6, while leaving IRE1α activity intact, again underscoring the IRE1α-independent effect on the proinflammatory responses initiated by ICD.

We then treated A375 cells with KIRA AfBP, followed by photocrosslinking to covalently modify protein targets and their pull-down (Fig. 6F). This approach unraveled the presence of several fluorescently labeled bands (Fig. 6G), indicating that KIRA6 AfBP could bind to different proteins. The intensity of all fluorescent bands decreased when the binding of KIRA6 AfBP was outcompeted by the presence of KIRA6 (Fig. 6G), demonstrating that proteins targeted by KIRA6 AfBP were effectively also bona fide KIRA6 targets. KIRA6 AfBP pull-downs were then subjected to on-bead tryptic digestion followed by mass spectrometry (MS). Table 1 lists the top 15 proteins (based on total spectrum counts) found to interact with KIRA6 AfBP.

**Table 1:** Putative KIRA6 off-target proteins.

Protein	Total spectrum count	% coverage
cDNA FLJ52842, highly similar to Actin, cytoplasmic 1	44	34%
cDNA FLJ59206, highly similar to Eukaryotic translation initiation factor 4B	35	17%
Nuclear ubiquitous casein and cyclin-dependent kinase substrate 1	32	25%
p180/ribosome receptor	26	6%
Transgelin-2	24	28%
Enolase 1, (Alpha)	21	27%
Triosephosphate isomerase	19	22%
Zinc finger Ran-binding domain-containing protein 2	18	25%
Heat shock 60 kDa protein 1	17	12%
Annexin A1	16	18%
Heat shock protein 90 kDa alpha (Cytosolic), class A member 1	16	17%
Heat shock protein 90 kDa alpha (Cytosolic), class B member 1	15	16%
Splicing factor, arginine/serine-rich 2	13	11%
Peptidylprolyl isomerase	13	4%
Proliferation-associated 2G4	12	10%

### The anti-inflammatory action of KIRA6 involves inhibition of HSP6_0_-NF-kB axis

Among the putative KIRA6 off-targets, we then focused our attention on the molecular chaperones HSP60 and HSP90 for different reasons: (i) except for actin, both molecular chaperones were the top-listed ATP-binding proteins identified by MS (Table 1), (ii) HSP90 is a known regulator of NF-κB-driven inflammatory signaling; [31, 33, 34], (iii) the cytosolic fraction of HSP60 has been recently shown to promote CXCL8 production by cancer cells by positively regulating NF-κB [35], and iv) no studies have linked intracellular HSP60 or HSP90 to ICD.

CDDP, MTX, or Hyp-PDT did not elevate the overall expression of HSP60 in A375 cells (Supplementary Fig. 7A) while CDDP and MTX, but not Hyp-PDT, reduced significantly the level of HSP90 (Supplementary Fig. 7B).

**Fig. 7:**
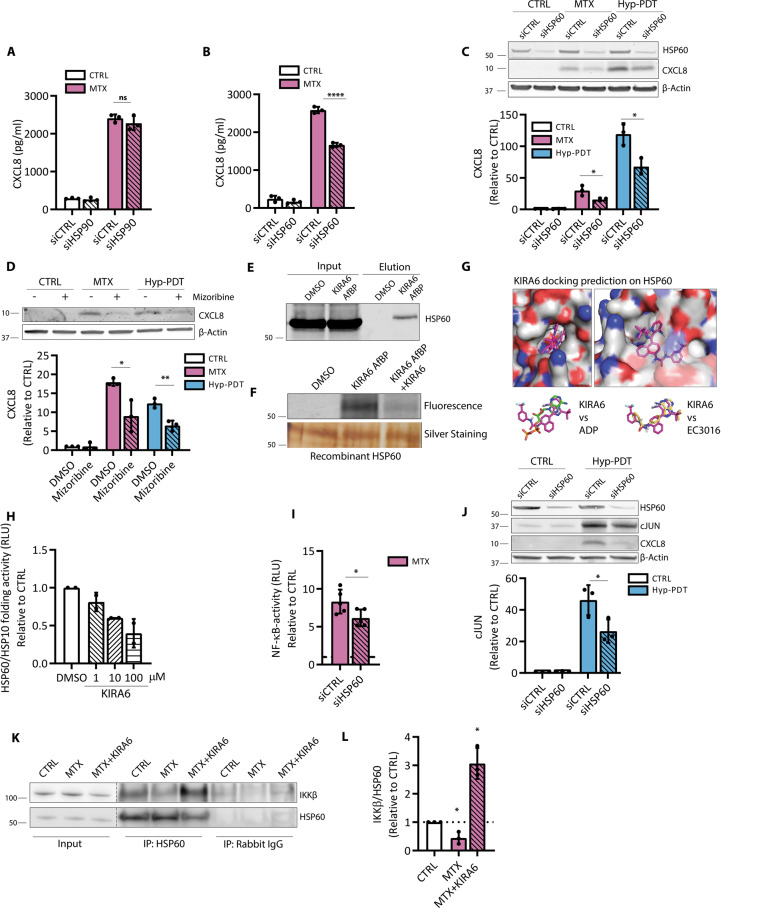
HSP60 is a KIRA6 target modulating CXCL8 production. **A** CXCL8 secretion measured by ELISA in conditioned medium from A375 cells upon siRNA-mediated HSP90 knockdown (siHSP90) or **B** HSP60 knockdown (siHSP60) compared to scramble siRNA (siCTRL) 24 h after treatment with MTX. **C** Effect of siHSP60 with respect to siCTRL on intracellular CXCL8 accumulation 4 h after treatment with MTX and Hyp-PDT. Data are expressed as fold change over control incubated with siCTRL. β-actin was used as loading control. **D** Impact of HSP60 inhibitor mizoribine (300 μM) on intracellular accumulation of CXCL8 4 h after treatment with MTX or Hyp-PDT. **E** Streptavidin-mediated pull-down of HSP60 with KIRA6 AfBP (10 μM) conjugated with biotin. **F** Recombinant HSP60 labeling with KIRA6 AfBP (10 μM) and with co-incubation KIRA6 AfBP (10 μM) and KIRA6 (100 μM). **G** KIRA6 docking prediction on HSP60 (PDB: 4PJ1) using Autodock Vina. This pose is compared with that obtained from docking of EC3016 (inhibitor of GroEL, prokaryotic ortholog of HSP60) and ADP into the same structure. **H** In vitro refolding activity of the HSP60/HSP10 chaperone complex after 1 h of incubation with heat-mediated unfolded substrate proteins in control condition or in the presence of KIRA6 at the indicated concentrations; *n* = 2 biological replicates/concentration. Data are expressed as fold change compared to control. **I** NF-κB activity measured by luciferase assay in A375 cells stably expressing the reporter 4 h after treatment of the siCTRL or siHSP60 transfected cells with MTX. Data are expressed as fold change compared to untreated cells transfected with the correspondent siRNA, whose reference value is indicated with a dotted line. **J** cJUN levels are measured in siHSP60 A375p with respect to siCTRL 4 h after treatment with Hyp-PDT. **K, L** Co-immunoprecipitation of IKKβ with HSP60 from the cytosolic fraction of A375 in basal condition or 2 h after treatment with MTX with or without co-incubation with KIRA6 (1 μM). In all graphs values are presented as mean ± SD of at least *n* = 3 independent biological replicates, except as reported in (**H**). Data are analyzed by two-tailed Student’s *t*-test in all the graphs except for one-sample *t*-test in (**L**) *p < 0.05, **p < 0.01,****p < 0.0001, ns not significant.

We then assessed whether silencing HSP60 or HSP90 (70–80% knockdown efficacy) (Fig. 7C, Supplementary Fig. 7C) could reproduce (at least in part) the effects of KIRA6 on the inhibition of CXCL8 production. While the knockdown of HSP90 had no major effects (Fig. 7A, Supplementary Fig. 7C) HSP60 silencing reduced CXCL8 secretion after MTX (Fig. 7B) and its intracellular accumulation after MTX and Hyp-PDT (Fig. 7C), without altering the fraction of dying cells (Supplementary Fig. 7D). Significant, albeit limited, decrease of CXCL8 and CXCL1 secretion was observed by HSP60 silencing in HeLa and CT26 (Supplementary Fig. 7E, F) cells. Likewise, co-incubation with mizoribine, a KIRA6 unrelated HSP60 inhibitor [36], reduced CXCL8 production following MTX or Hyp-PDT (Fig. 7D).

While these results do not completely rule out the contribution of HSP90, they suggest a causal link between the anti-inflammatory action of KIRA6 and HSP60.

Congruently, KIRA6 AfBP pulled down HSP60 from the lysate of A375 cells (Fig. 7E) and its binding to recombinant HSP60 was competitively inhibited by the presence of KIRA6 (Fig. 7F).

KIRA6 shares structural similarity with two reported ATP-competitive inhibitors of HSP60 [37] and GroEL [38] (prokaryotic ortholog of HSP60). Docking simulation using Autodock Vina [39] against human HSP60 (PDB: 4PJ1 [40]) showed that while the aromatic amine of the imidazopyrazine ring in KIRA6 faces the opposite direction compared with the amine of the adenine ring (ATP molecule crystallized with HSP60), we observed a good overlap between the two rings as well as good alignment with two proton acceptors (Fig. 7G). A calculated hydrogen bond interaction between HSP60 and KIRA6 (within 3 angstroms) is indicated (Supplementary Fig. 7G). This pose was also in excellent alignment with the docking of EC3016 (GroEL inhibitor) into the same structure, with comparable binding energies (Supplementary Fig. 7H).

HSP60-mediated protein folding requires ATP binding and hydrolysis [41]. HSP60 folding ability was reduced by KIRA6 (Supplementary Fig. 7I), in a concentration-dependent manner (Fig. 7H) suggesting that the HSP60-KIRA6 complex exhibits impaired ATPase and folding ability.

We then explored the mechanism by which KIRA6 could inhibit CXCL8 production by targeting HSP60. HSP60 silencing decreased NF-κB activity and cJUN upregulation upon treatment with MTX or Hyp-PDT, respectively (Fig. 7I, J), suggesting that HSP60 is an upstream regulator of these proinflammatory pathways.

HSP60 is an abundant mitochondrial chaperonin with recently reported extramitochondrial activities [37]. Immunofluorescence and immunoblot analysis (Supplementary Fig. 7J, K) did not reveal a detectable intracellular redistribution of HSP60 after CDDP, MTX, or Hyp-PDT treatments. Also we found no significant upregulation of mitochondrial unfolded protein response (mtUPR)-related genes [38] (Supplementary Fig. 7L). These data suggest that the mitochondrial pool of HSP60 was unlikely to be involved in the pathway leading to CXCL8 production after ICD.

Whereas no previous study linked HSP60 to cJUN upregulation, recently HSP60 has been shown to contribute to TNF-α-mediated NF-κB activation via direct interaction with the IKK complex in the cytosol [35]. Co-IP analysis revealed that in unstressed cells a fraction of HSP60 was found in a complex with IKKβ (Fig. 7K). MTX treatment attenuated the binding of HSP60 to IKKβ (Fig. 7K, L) while the co-treatment of MTX with KIRA6 enforced the HSP60-IKKβ complex interaction (Fig. 7K, L). While the mechanistic aspects of this interaction require further explorations, these findings suggest that KIRA6 by interfering with the folding activity of HSP60 locks the cytosolic HSP60-IKKβ complex in an inactive conformation and/or prevents IKKβ to dock to other partner proteins [42], thus compromising NF-κB signaling.

## Discussion

**Fig. 8:**
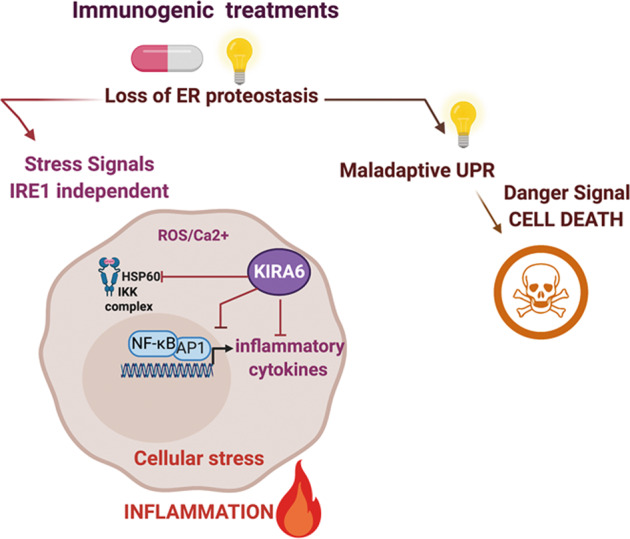
KIRA6 curtails the inflammatory traits of immunogenic treatments. Chemotherapy with mitoxantrone or Hypericin-mediated photodynamic therapy (Hyp-PDT) (illustrated by the light as essential triggering factor) induces loss of ER homeostasis, which leads in case of the most pronounced ER stress inducer Hyp-PDT to maladaptive UPR and immunogenic cell death. Both immunogenic treatments, however, also elicit an early stress response, which is independent of the UPR/ISR and caspase-mediated cell death. This premortem stress response leads to the production of a common subset of proinflammatory chemokines through ROS and Ca^2+^-mediated activation of the NF-κB and AP-1 transcriptional program. KIRA6, an inhibitor of the IRE1α kinase activity, blunts the inflammatory output of immunogenic therapies in an IRE1α-independent manner. One of the off-target effectors of KIRA6 is the cytosolic HSP60, which is required for the full activation of NF-κB/AP-1 and CXCL8 production by immunogenic treatments.

In this study, by performing RNA-seq analysis and signaling pathway validation we portray a NF-κB/AP-1-driven inflammatory trait, shared by stressed/dying cancer cells responding to two paradigms of immunogenic treatments. This common proinflammatory pathway segregates from the activation of the UPR induced by these anticancer treatments and proceeds unrelated to caspase-induced cell death. We show that the IRE1α inhibitor KIRA6 abolishes the inflammatory output associated with immunogenic treatments through IRE1α-independent mechanisms, which involves HSP60 modulation of NF-κB and cJUN-driven CXCL8 induction (Fig. 8).

The causal link between the UPR pathway and sterile inflammation has been elucidated in several studies [43, 44] using both genetic and pharmacological approaches, which provided independent support for the role of the PERK and IRE1α pathways in NF-κB activation.

Canonical ER stress agents, such as thapsigargin or tunicamycin, or certain chemotherapies such as taxanes, stimulate the PERK/ATF4/CHOP-dependent upregulation of DR5 [45–48], leading to ligand-independent NF-κB-mediated proinflammatory signaling [6]. Our study, however, shows that this is not a general ‘modus operandi’ of all agents eliciting ER stress. Here, loss of ER proteostasis induced by MTX and prevalently Hyp-PDT [3, 22] promotes a rapid NF-κB and AP-1 activation as premortem stress responses stimulated by Ca^2+^ and ROS-induced signals, which precedes apoptosis. Upregulation of DR5 and caspase signaling are dispensable for the transcriptional upregulation of CXCL8 induced by MTX and Hyp-PDT. Thus, a differential involvement of the CHOP-DR5 pathway could be inherent in the apical triggers causing loss of ER homeostasis and consequent UPR or ISR.

Irrespective of this possibility, our study shows that only IRE1α kinase inhibitor, KIRA6, can overrule the proinflammatory output of immunogenic treatments. In MTX-treated cancer cells, KIRA6 inhibited NF-κB-driven transcription of several cytokines/chemokines, their chemoattractant and DC-maturation ability and significantly reduced the vaccination potential of MTX-treated cancer cells in syngeneic mice, without altering, if anything even increasing, DAMP exposure. Together these results unravel that the proinflammatory output of chemotherapy-treated cancer cells is a crucial component of their in vivo immunogenicity. Further research is required to evaluate the impact of blocking the proinflammatory output of ICD in curative settings.

KIRA6 is a prototype of ATP-binding compounds disrupting the oligomerization and RNase activities of IRE1α [27, 49], which has been used as specific in vitro and in vivo inhibitor of the IRE1α proinflammatory branch of the UPR [44, 49, 50]. Here, using IRE1α deficient cells and a KIRA6-clickable photoaffinity probe [32], we found that IRE1α was dispensable for the anti-inflammatory ability of KIRA6 and identified HSP60 and HSP90 as potential off-targets of this inhibitor.

HSPs respond to loss of proteostasis by exerting various intracellular cytoprotective functions and by regulating inflammatory and immune responses [51] and HSP90 inhibitors such as geldanamycin and radicicol are promising anticancer therapeutics [51]. In contrast, a role for the cytosolic pool of HSP60 in the NF-κB pathway has only recently emerged [35]. While further investigations are required to fully appreciate the mechanistic underpinnings of the inhibitory effects of KIRA6, our study highlights a preferential role for HSP60 in the proinflammatory pathway initiated by ICD. By targeting cytosolic HSP60 and reducing its folding activity, KIRA6 likely locks the HSP60-IKKβ complex in a conformation with a reduced ability to activate NF-κB-mediated proinflammatory responses.

In conclusion, we show that two established paradigms of ICD share a common transcriptional signature, involving a proinflammatory early stress response operating in parallel to the UPR, which contributes to the in vivo immunogenicity of MTX. This early proinflammatory response involves HSP60 and can be overruled by KIRA6, independently of IRE1α. Considering that small molecule inhibitors of the IRE1α pathway have been proposed as potential anticancer therapeutics [52], our study also raises caution about the use of KIRA6 to assess the role of IRE1α in inflammatory pathologies.

## Materials and methods

### Chemical inhibitors

Brefeldin A, SCH772984, 4μ8C, KIRA6, and mizoribine were purchased by Selleckchem. BAY11–7082, GSK2606414, STF-083010 were purchased by Caymanchem). N-Acetyl-L-cysteine and L-Histidine were supplied by Sigma–Aldrich, thapsigargin by Enzo Life Sciences, BAPTA-AM by ThermoFisher Scientific. Bortezomib was obtained directly from the Pharmacy Department of the Leuven University Hospital, Leuven Belgium.

### Cell culture and treatments

A375 were cultured in DMEM (Sigma–Aldrich) supplemented with 2 mM glutamine (Sigma–Aldrich) and 10% fetal bovine serum (PAN-Biotech); CT26, MEF, HeLa, HCT-116, THP1, and HEK293T were cultured in DMEM supplemented with 1 mM glutamine and 10% FBS. All cell lines were cultured at 37 °C under 5% CO2. To induce cell death A375, HeLa, MEF, and HCT-116 cells were incubated with 1 μM mitoxantrone (MTX, Sigma–Aldrich) or 50 μM Cisplatin (CDDP, Sigma–Aldrich). CT26 were incubated with 5 μM MTX or 50 μM CDDP. For hypericin-based photodynamic therapy (Hyp-PDT) conditions, A375 cells were incubated for 16 h with 150 nM hypericin (Enzo Life Sciences) in full medium followed by removal of hypericin, irradiation (2.70 J/cm^2^) and cultured for indicated times. Chemical inhibitors were preincubated for 1 h before addition of cell death inducers and maintained in the medium, unless specified otherwise. In the absence of cell death induction, chemical inhibitors of signaling pathways, Ca^2+^ chelators or ROS quenchers, did not alter the low/baseline levels of chemokines production by the untreated cancer cells, therefore in some graphs these additional controls have been omitted. All cell lines were routinely screened for mycoplasma contamination. Human cell lines have been recently authenticated by STR profiling.

### Cell death assay

For cell death kinetics, cells were treated with cell death inducers in presence or absence of 50 μM z-VAD-FMK (Bachem) in medium containing 1 μM sytox green (Thermofisher Scientific). At the indicated time post-treatment, fluorescence emission at λ = 530 nm was measured at flexstation 3 (Molecular Devices). Cells were then lysed with Cell lysis buffer (Bioke) for 10 min at room temperature (RT) and fluorescence relative to 100% cell death was measured and used as normalization parameter. For other cell death assays, cells were collected 24 h after treatment with the cell death inducers with TrypLE Express (Life Technologies) and resuspended in PBS containing 0.5% bovine serum albumin (BSA, Sigma–Aldrich) and 1 μM sytox green and analyzed by flow cytometry on Attune (Thermofisher Scientific).

### Ecto-calreticulin detection

After treatment, cells were collected with TrypLE Express (Life Technologies), washed with PBS and with Flow cytometry (FC) buffer (3% BSA in PBS), incubated for 30 min at 4 °C with fluorophore-conjugated primary antibody (EPR3924, AbCAM), washed with FC buffer and resuspended in FC buffer including viability die and analyzed on Attune (Thermofisher Scientific). The permeabilized cells were excluded from the analysis due to intracellular staining.

### ATP assay

A375 cells were treated as indicated in 2% FBS medium. Extracellular ATP was measured in the conditioned with ATP Bioluminescent assay kit (Sigma–Aldrich) following manufacturer’s instructions. Bioluminescence was assessed by optical top reading via FlexStation 3 microplate reader (Molecular Devices).

### Immunofluorescence

Cells were plated on coverslips precoated with 0.1% gelatin, treated for the indicated time and fixed in 4% PFA. Cells were permeabilized 10 min with 0.1% Triton in PBS, then blocked for 1 h in blocking buffer (5% FBS, 1% BSA). Primary antibody was added in blocking solution and incubated overnight at 4 °C. After washing with TBS-T buffer (50 mM Tris, 150 mM NaCl, 0.1% Tween-20), coverslips were incubated with secondary antibody for 2 h at RT. Antibodies used are listed in Supplementary Table 1. Nuclei were counterstained with DAPI in PBS (1 μg/ul, 1:1000) and mounted on slides with Prolong Gold. Pictures were taken at Zeiss LSM 780 confocal microscope. For the representative image, the central stack was selected using ImageJ software.

### Western blot

Whole-cell lysates were loaded on 4–12% Bis-Tris gel and separated by SDS-PAGE on the Criterion system (Bio-Rad Laboratories), and electrophoretically transferred to nitrocellulose membrane. The blots were blocked for 1 h at RT with in TBS-T buffer containing 5% nonfat dry milk, and incubated with the indicated primary antibodies overnight at 4 °C in TBS-T containing 5% BSA. After washing with TBS-T, membranes were incubated with secondary antibodies conjugated with infrared fluorophores for 2 h at RT. The membranes were visualized with Typhoon biomolecular imager (Amersham). Alternatively, horseradish peroxidase secondary antibodies were used and the membranes were incubated with enhanced luminol-based chemiluminescent substrate (Amersham) and visualized with Amersham Imager 600. All antibodies used are listed in Supplementary Table 1.

### RNA extraction and qPCR

After treatment for the indicated time, total RNA was extracted from cells using TriSure buffer (Bioline) followed by phenol/chloroform extraction and 1 μg of RNA was reverse transcribed with Quantitect RT kit (Qiagen) following manufacturer’s instructions. Primers for real-time PCR were designed with the Primer3 web tool (Supplementary Table 2). The housekeeping 18 S ribosomal RNA was used to normalize the expression levels. Quantitative PCR (qPCR) was performed with ABI 7500 Fast Real-Time PCR System using ORA™ qPCR Green Master Mix (highQu).

### XBP1 splicing assay

The following primers were used: unspliced XBP1 (5’-CAGCACTCAGACTACGTGCA-3’, sense), spliced XBP1 (5’-CTGAGTCCGAATCAGGTGCAG-3’, sense), unspliced and spliced XBP1 (5’- ATCCATGGGGAGATGTTCTGG-3’, antisense). The RT-PCR analyses were performed according to the following conditions: denaturation at 95 °C for 10 min, followed by 35-cycles of denaturation at 95 °C for 10 seconds, annealing at 5 °C for 30 seconds, extension at 7 °C for 1 min, and final extension at 72 °C for 7 min. Amplicons were resolved in 2% agarose gels.

### ELISA

Eight or 24 h after treatment, conditioned medium was collected and the levels of secreted human CXCL8 and murine CXCL1 were detected with DuoSet ELISA (R&D systems) following the manufacturer’s instructions. Absorbance values were measures on Flexstation 3 at λ =  450 nm and absorbance background values at λ = 540 nm were subtracted.

Multiplexed ELISA was performed on a Luminex FLEXMAP 3D® platform (Luminex, Austin, TX), using a custom-developed chemokine 6-plex panel (ProtATonce, Athens, Greece). Custom antibody-coupled beads were technically validated as described before [53].

### SiRNA transfection

Cells were transfected by adding 1 ml serum-free culture media with Trans-IT X2 transfection reagent (MirusBio) and targeting (On-Target Smartpool siRNA) or scrambled (siCTRL) siRNA (Dharmacon, Thermo Fisher Scientific) twice on two consecutive days. Experiments were performed 48 h after the second transfection.

### NF-κB activity

Plasmid pHAGE NFkB-TA-LUC-UBC-GFP-W containing the luciferase gene under the minimal NF-κB promoter was a gift from Darrell Kotton (Addgene plasmid #49343; http://n2t.net/addgene:49343; RRID:Addgene_49343). To generate lentiviral particles, HEK 293 T cells were transfected with pHAGE plasmid in the presence of plasmid encoding VSV-G (pMD2-VSV-G, Tronolab) and packaging proteins (pCMVdR8.9, Tronolab). Twenty-four hours after transfection transfecting medium was substituted with fresh medium and VSV-G pseudotyped virus was collected 48 h after transfection and added to the exponentially growing A375 cell cultures in the presence of hexadimethrine bromide (Sigma–Aldrich). Cells were expanded and GFP positive cells were sorted with Influx (BD Biosciences). Four hours post-treatment, NF-κB activity was measured with luciferase assay kit (BioAssay System) following manufacturer’s protocol at Flexstation 3.

### Chemotaxis

Human neutrophils were obtained from fresh human peripheral blood from healthy volunteers and were isolated using a Percoll (Sigma–Aldrich) gradient.

Conditioned supernatants were generated from treated A375 cells for 24 h in DMEM without FBS. Conditioned supernatants were then deprived of chemical inhibitors by using Amicon® Centrifugal Filter Unit and washed with two volumes of PBS and resuspended to 10× the initial concentration. Chemotaxis assays were performed with Transwell®polycarbonate membrane cell culture inserts (Corning). Hundred microliters of 10-times concentrated supernatant diluted in 400 μL of DMEM were added to the bottom well of a chemotaxis chamber and 5 × 10^4^ THP1 cells or primary human peripheral blood neutrophils were added. For rescue experiments, CXCL8 and CXCL8-neutralizing antibody were added to the bottom well at a concentration of 10 ng/ml and 0.5 μg/ml, respectively. Total cells entering the bottom chamber were counted after 2 h and representative pictures were taken at Olympus IX73 inverted microscope (Olympus Life Sciences).

### DC-maturation analysis

Human immature DCs (iDC) were prepared according to previously described protocols [54]. The iDCs were cocultured for 24 h at a 1:20 (DCs:cancer cells) ratio with lysates generated by three freeze/thaw cycles from untreated or dying cancer cells (24 h timepoint after treatment). Alternatively, iDCs were cultured for 24 h in the presence of the ‘cell-free’ supernatant generated from treated A375 cells for 24 h in DMEM without FBS, deprived of chemical inhibitors as described above. After 24 h, DCs were stained with anti-HLA-DR (LN3, Biolegend) and anti-CD86 (IT2.2, Biolegend) and analyzed by FACS.

### In vivo prophylactic vaccination

BALB/c female mice aged 6–8 weeks were injected subcutaneously (twice, with a 7 days interval) with 100 μL containing 1 × 10^6^ dying CT26 cells, or with 100 μL of PBS into the flank. After 10 days, mice were rechallenged with 5 × 10^5^ untreated CT26 cells into the opposite flank. Tumor growth was measured with an electronic digital caliber and monitored for 50 days, and mice were euthanized by cervical dislocation when tumor size reached 1500 mm^3^. Tumor volume (mm^3^) was calculated with the following formula: (tumor length − 0.5) × (tumor width − 0.5) × (tumor depth − 0.5)*(π/6).

### IRE1 knockout generation

A375 IRE1 knockout and scramble control were generated with CRISPR-Cas9 double nickase system (Santa Cruz Biotechnology) according to manufacturer’s protocol.

### KIRA6 AfBP generation and target labeling

KIRA6 AfBP was generated as previously reported [32].

A375 whole-cell lysates were normalized to a concentration of 1 mg/mL in a volume of 30 μL. Samples were then treated with KIRA6 AfBP (10 μM) or DMSO, mixed by vortexing, and immediately irradiated for 6 min at RT. For competition experiments, samples were co-treated with KIRA6 AfBP (10 μM) and KIRA6 (100 μM). For HSP60 labeling, 160 ng of recombinant human HSP60 were used.

After irradiation, probes were clicked onto TAMRA-azide (Carl Roth) using the following conditions: 25 μM of tag-azide, 50 μM of THPTA (Sigma–Aldrich), 1 mM of CuSO4 (freshly prepared) and 1 mM of sodium ascorbate (freshly prepared). Click reaction was incubated for 1 h at RT and the reaction was quenched by addition of 10 μL of 4× SDS loading buffer. Samples were resolved by 10% SDS-PAGE. Following visualization, gels were stained with coomassie using ROTI®Blue (Carl Roth).

### Mass spectrometry and data analysis

Live A375 cells were incubated with KIRA6 AfBP (10 μM) for 1 h and irradiated for 6 min at RT. Proteins were then extracted and probes were clicked onto azide-functionalized magnetic beads (Jena Bioscience) as described above. Probe-labeled proteins were enriched with magnetic isolation and extensively washed. To perform disulfide bonds reduction and cysteine alkylation, the beads were resuspended in denaturation buffer (7 M urea, 20 mM HEPES) and DTT (1 mM) was added for 45 min at room temperature. Then iodoacetamide (4 mM) was added and incubated for 45 min at room temperature. Finally, DTT (5 mM) was added for 45 min at room temperature to quench the remaining iodoacetamide. On-bead trypsin digestion was executed overnight at 37 °C in the presence of 0.6 μg of trypsin, 200 mM ammonium bicarbonate, 2.5 % acetonitrile, and 0.005 % ProteaseMax. The resulting peptide mixture was subjected to C18 Zip Tip clean-up (Millipore) before being analyzed by high-resolution LC-MS/MS using an Ultimate 3000 Nano Ultra High Pressure Chromatography (UPLC) system interfaced with an orbitrap Elite mass spectrometer via an EASY-spray (C18, 15 cm) column (Thermo Fisher Scientific). Peptides were identified by MASCOT (Matrix Science) using the Homo sapiens database (173330 entries), adopting the following MASCOT search parameters: trypsin, two missed cleavages allowed, oxidation (M) was specified as variable modification, carbamidomethylation of cysteine was specified as fixed modification. Mascot was searched with a fragment ion mass tolerance of 0.50 Da and a parent ion mass tolerance of 10 ppm. Scaffold software was used to validate MS/MS based peptide and protein identifications, being accepted if they could be established by a probability greater than 95% and 99%, respectively. The presence of at least two unique identified peptides per protein was required.

### Off-target validation by pull-down

After co-incubation with A375 protein lysates and irradiation,  KIRA6 AfBP was clicked onto TAMRA-azide-PEG-biotin as described above. The excess reagents from the samples were then removed by acetone precipitation. Following resuspension of the pellets to a final volume of 100 μL, half of the sample was kept as the input control. The remaining 50 μL were incubated with 20 μL of prewashed streptavidin beads (ThermoFisher) for 1 h with mixing at RT. The supernatant was removed and beads were sequentially washed with 0.33% SDS in PBS, 1 M NaCl and PBS. Bound proteins were eluted with sample buffer (62.5 μM Tris-HCl, 10% glycerol, 2% SDS, 1× protease inhibitor, 1× phosphatase inhibitor) and resolved by western blot.

### Molecular docking

The crystal structure of human HSP60 in complex with ADP (PDB: 4PJ1 [55]) was used as the template for docking of KIRA6 and EC3016. The protein structure for docking was prepared using AutoDockTools 1.5.6 [56]. KIRA6 and EC3016 input files were drawn in Chemdraw Prime 19.0, energy-minimized, converted to PDB files using Chem3D Prime 19.0 and converted into a PDBQT files by AutoDockTools [57]. The docking simulation was performed with AutoDock Vina 1.5.6. The visualization of the results was done using PyMOL molecular viewer.

### RNA-seq and bioinformatics analysis

Raw RNA-seq FASTQ files were preprocessed to remove technical artifacts, performing quality trimming to trim low-quality ends (<Q20) and remove trimmed reads shorter than 35 bp using FastX 0.0.14 [58], adapter trimming (considering at least 10 bp overlap and 90% match) with cutadapt 1.7.1 [59], and quality filtering using FastX 0.0.14 and ShortRead 1.24.0 to remove polyA-reads, ambiguous reads containing N’s, low-quality reads (with more than 50% of the bases < Q25) and artifact reads (with all but three bases in the read equal one base type). RNA-seq reads were aligned to the Homo sapiens GRCh3773 reference genome using STAR 2.4.1d [60], with the following parameter settings: --outSAMprimaryFlag OneBestScore –twopassMode Basic --alignIntronMin 50 --alignIntronMax 500000 --outSAMtype BAM SortedByCoordinate. The samtools 1.1 toolkit was used to remove reads with non-primary mappings or with a mapping quality ≤ 20 [61] and for BAM/SAM file sorting and indexing. Gene counts were computed with featureCounts 1.4.6 [62], using the following options: -Q 0 -s 2 -t exon -g gene id.

Differential expression analysis was performed with the R package edgeR [63], considering only protein-coding genes with at least one count-per-million (CPM) in not less than three samples. Gene counts were normalized between samples with trimmed-mean of M-values (TMM) normalization [64] and dispersions were estimated with the Cox-Reid profile-adjusted likelihood method [65]. Given the small number of replicates, the quasi-likelihood F-test was used for testing [66]. Genes differentially expressed in the treated cell lines with respect to the control at any timepoint were selected using a level of significance of 0.1 on *p* values adjusted for multiple testing with the Benjamini–Hochberg approach and imposing a cutoff of one on absolute log-fold-changes.

Principal component analysis was performed on the 10% most variable genes considering rlog-normalized counts computed with the DeSeq2 R package [67]. Gene Ontology (GO) was performed with the R package *TopGO* [68] using as input the significantly upregulated genes compared to time-matched untreated control separately by each treatment at each timepoint analyzed and using the entire gene list mapped by RNA-seq as reference library. The gene ontology annotations relative to Biological Processes and Cellular Compartments were provided by the *org.Hs.eg.db* and *GO.db* annotation packages. Gene Set Enrichment Analysis (GSEA) [16] was perform on WebGestalt [69] against Wikipathway Cancer database submitting as entry the output from PCA with genes weighed for their contribution to the variance of the first two principal components. Transcription factor prediction was performed with Ingenuity Pathway Analysis (Qiagen), iRegulon [70] Cytoscape plugin and TRANSFAC annotation-based GATHER [71] tool submitting as entry the list of genes belonging to the GO Cellular Component term “extracellular space” that were jointly upregulated by Hyp-PDT and MTX treatments.

### Isolation of subcellular fractions

Cytosol and mitochondria were separated from A375 cells 2 h or 4 h after treatment using the Mitochondria/Cytosol Fractionation Kit (Abcam) following the manufacturer’s instructions.

### Co-immunoprecipitation

Cytosol was isolated as described above from A375 2 h after treatment and 500 μg of proteins were combined with primary antibody overnight at 4 °C. Protein–antibody complexes were captured by addition of Protein AG Magnetic Beads (Pierce) for 1.5 h at RT. Protein AG Magnetic Beads with captured protein–antibody complexes were washed three times with lysis buffer. Proteins were eluted with sample buffer (62.5 μM Tris-HCl, 10% glycerol, 2% SDS, 1× protease inhibitor, 1× phosphatase inhibitor) and loaded on gel for western blot analysis.

### HSP60 refolding assay

Protein refolding efficiency of HSP60/HSP10 chaperone complex was assessed with Human HSP60/HSP10 Protein Refolding Kit (Biotechne) following manufacturer’s instructions.

### Statistical analysis

Data are presented in fold changes, absolute values, or percentages with mean ± SD. as indicated in figure legends. All statistical analyses were performed using Prism software (GraphPad Software, USA) and indicated in the figure legends.

## Supplementary information

Supplementary Figures and Tables

## Data Availability

The experimental data from RNA sequencing are available in the NCBI Gene Expression Omnibus (GEO) repository (https://www.ncbi.nlm.nih.gov/geo/) with accession number GSE163377.
