# Supplementary material for: Stress-induced inflammation evoked by immunogenic cell death is blunted by the IRE1α kinase inhibitor KIRA6 through HSP60 targeting
Source: Cell Death Differ. 2021 Aug 27;29(1):230–45. doi: 10.1038/s41418-021-00853-5 (PMC8738768; doi:10.1038/s41418-021-00853-5)
Supplement: Supplementary file 1 — Supplementary Figures and Tables [file 41418_2021_853_MOESM1_ESM.pdf]

SUPPLEMENTARY INFORMATION

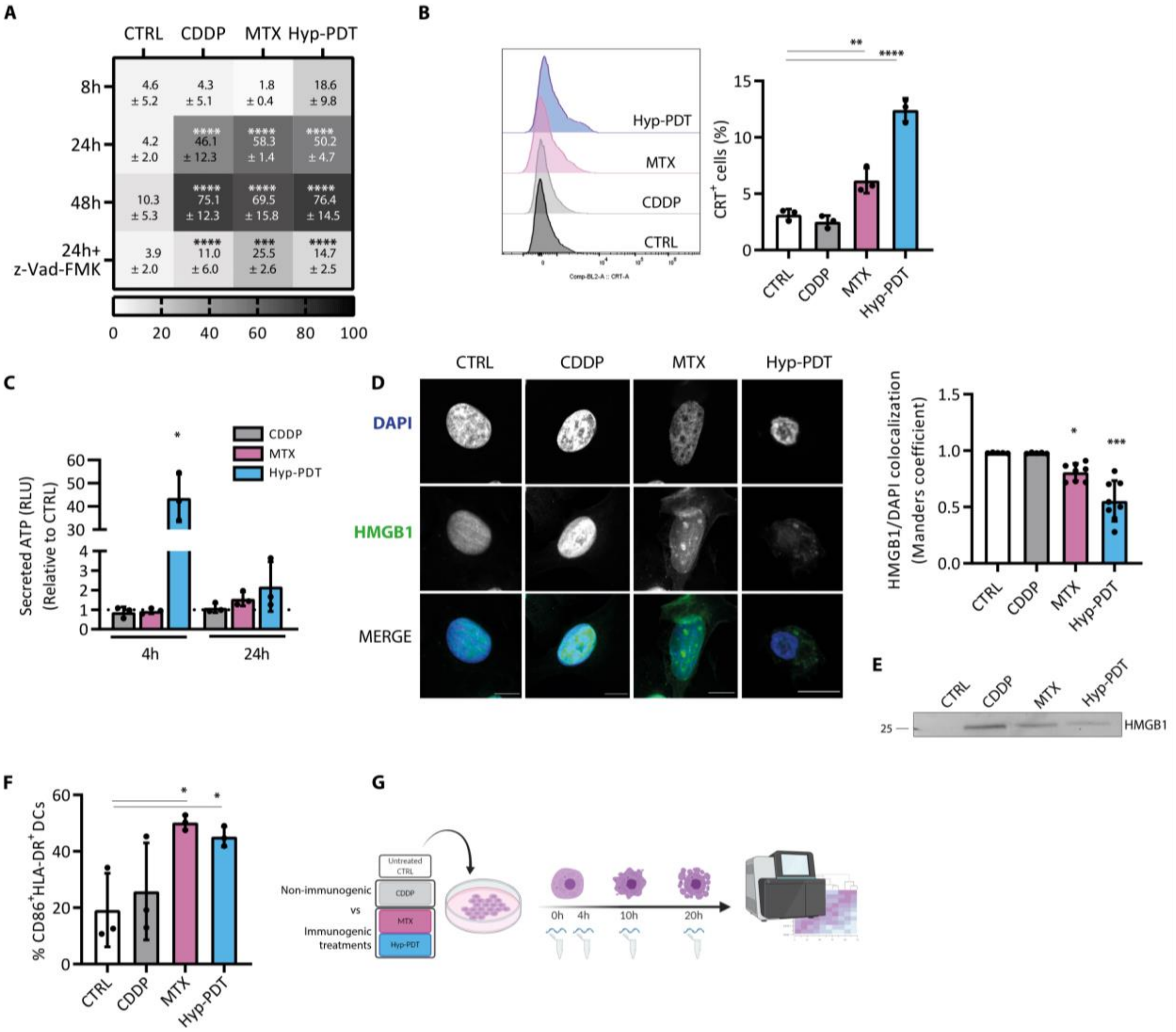

Supplementary Figure 1 (A) Heatmap summarizing percentage of cell death measured at the indicated time points after treatment of A375 cells with CDDP, MTX or Hyp-PDT detected by the uptake of Sytox Green. The impact of the pan-caspase inhibitor z-Vad-FMK (50 μM) on cell death in basal condition or induced by CDDP, MTX, or Hyp-PDT was assessed 24 h after treatment. **(B)** A375 cells treated with CDDP, MTX, or Hyp-PDT were evaluated for the externalization of CRT in non-permeabilized cells by FACS staining 4 h after treatment. Dead cells were excluded from the analysis. **(C)** ATP secretion in the medium was measured by luciferase-based assay at the indicated time points after treatment with CDDP, MTX, or Hyp-PDT. **(D)** Representative confocal microscopy images for the intracellular redistribution of HMGB1 (Green) in A375 cells 16 h after treatment with CDDP, MTX, or Hyp-PDT. Nuclei were counterstained with DAPI (blue). Scale bar: 10 μm. Correlation of DAPI and HMGB1 colocalization is analyzed with Manders coefficient. **(E)** Representative Western Blot for HMGB1 secretion in the medium from A375 24 h after treatment with CDDP, MTX, or Hyp-PDT. **(F)** Dendritic cell maturation was measured by upregulation of CD86 and HLA-DR by FACS 24 h after coincubation with whole cell lysate of A375 cells treated for 24 h with CDDP, MTX, or Hyp-PDT. **(G)** Schematic representation of the experimental setup utilized for the RNAseq.

In all graphs values are presented as mean ± SD of at least n=3 independent biological replicates. Data is analyzed by two-way ANOVA followed by Dunnett's multiple comparison test in (A), one-way ANOVA followed by Dunnett's multiple comparison test in (B, D, F) and one sample t-test in (C), *p<0.05,**p<0.01, ***p<0.001, ****p<0.0001 versus time-matched control, $p<0.05, $$p<0.001 versus time matched-treatment.

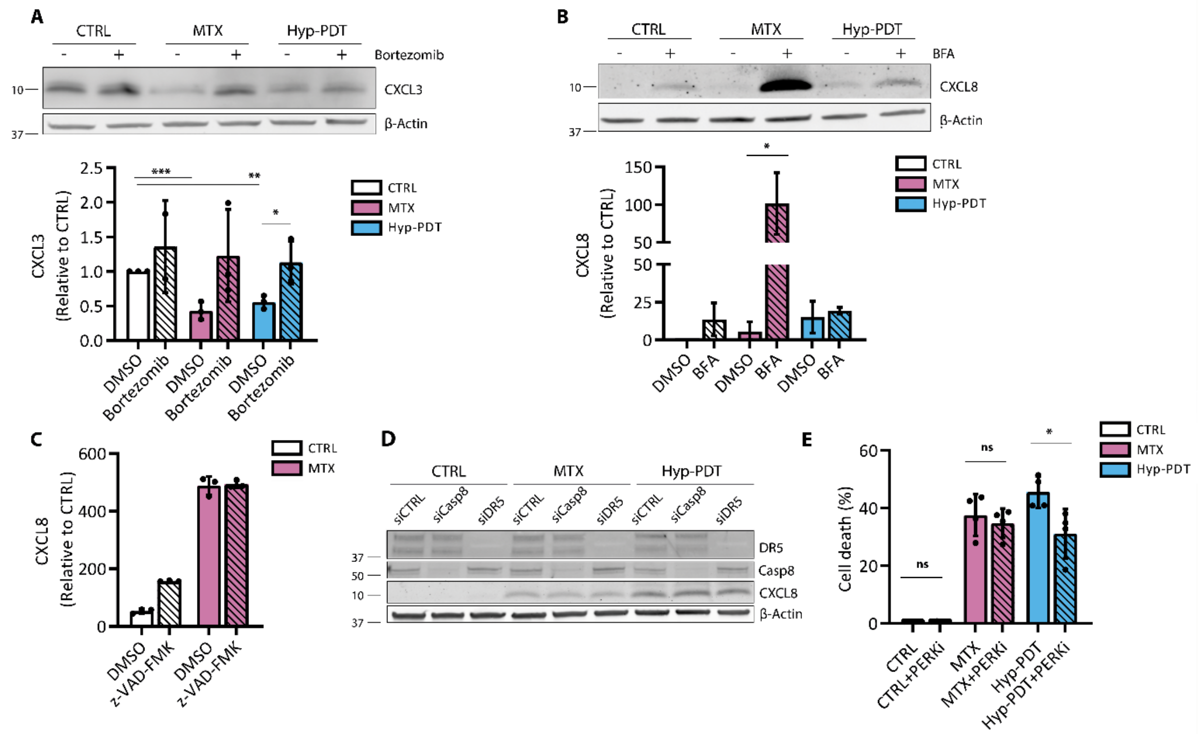

Supplementary Figure 2 (A) Intracellular CXCL3 levels were assessed 8 h after treatment with MTX or Hyp-PDT in the presence or absence of the proteasome inhibitor Bortezomib (150 nM). **(B)** Intracellular CXCL8 levels were assessed 24 h after treatment with MTX or Hyp-PDT in the presence or absence of Brefeldin A (BFA, 50 ng/ml). **(C)** Extracellular CXCL8 levels were measured by ELISA in the supernatant of HeLa cells 24 h after treatment with MTX in the presence or absence of z-VAD-FMK (50 μM). **(D)** Impact of siDR5 and siCasp8 on CXCL8 protein production measured by Western Blot 4h after treatment with MTX or Hyp-PDT compared to scrambled siRNA (siCTRL). **(E)** Cell death was assessed 24 h after treatment with MTX and Hyp-PDT in the presence of absence of PERK inhibitor GSK2606414 (1 μM).

In Western Blots β-actin was used as loading control. In all graphs values are presented as mean ± SD of at least n=3 independent biological replicates**.** Data is analyzed by two-tailed Student's t-test in all graphs and One-way ANOVA versus CTRL followed by Dunnett's multiple comparison test in (A),$^{*}p<0.05$ $^{**}p<0.01$, $^{***}p<0.001$, ns=not significant.

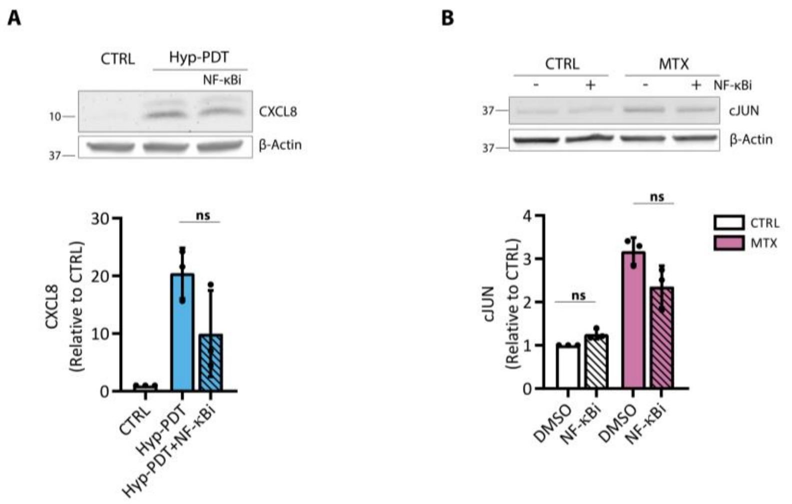

Supplementary Figure 3 (A) Intracellular CXCL8 levels were assessed 4 h after treatment with Hyp-PDT in the presence or absence of NF-κB inhibitor BAY11-7082 (10 μM). **(B)** Intracellular levels of cJUN were evaluated 4 h after treatment with MTX in the presence or absence of NF-κB inhibitor BAY11-7082 (10 μM).

In all Western Blots β-actin was used as loading control.

Data is presented as mean ± SD and analyzed by two-tailed Student's t-test, ns=not significant.

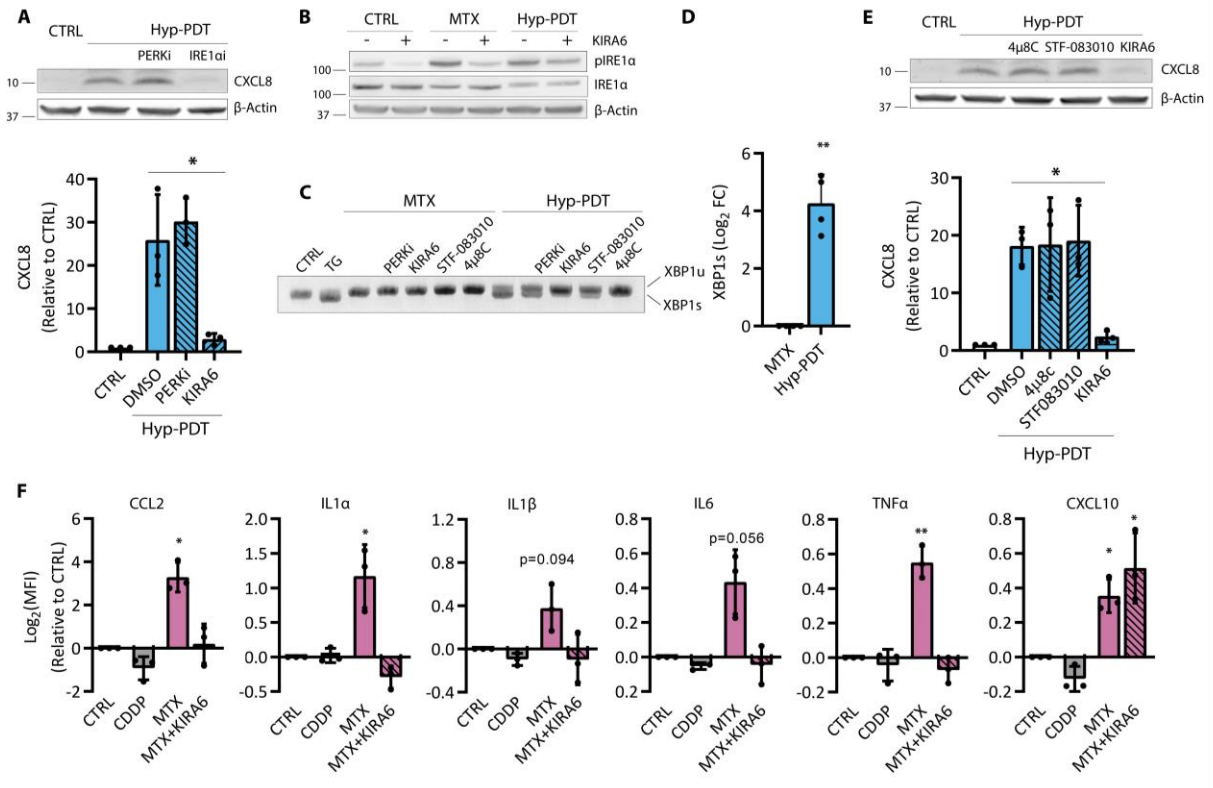

Supplementary Figure 4 (A) Intracellular CXCL8 levels were assessed 4 h after treatment with Hyp-PDT in the presence or absence of the PERK inhibitor GSK2606414 (1 μM) or the IRE1α inhibitor KIRA6 (1 μM). **(B)** Representative Western blot evaluating the efficacy of the IRE1α kinase inhibitor KIRA6 (1 μM) in inhibiting phosphorylation of IRE1α and intracellular CXCL8 accumulation 4 h after treatment with MTX or Hyp-PDT. **(C)** XBP1 splicing assay was performed by PCR 4 h after treatment with MTX or Hyp-PDT in the presence or absence of the PERK inhibitor GSK2606414 (1 μM) or the IRE1α inhibitors 4μ8C (100 μM), STF-083010 (50 μM) and KIRA6 (1 μM). The upper band refers to unspliced XBP1 (XBP1u) and the lower band to spliced XBP1 (XBP1s). Thapsigargin (TG, 2 μM) was used as positive control for XBP1 splicing. **(D)** mRNA levels of cleaved XBP1 (XBP1s) were measured by qPCR in A375 cells 4 h after treatment with MTX or Hyp-PDT. **(E)** Intracellular CXCL8 levels were assessed 4 h after treatment with Hyp-PDT in the presence or absence of the IRE1α RNAse inhibitors 4μ8C (100 μM) and STF-083010 (50 μM) and IRE1α kinase inhibitor KIRA6 (1 μM). **(F)** Cytokines and chemokine secretion in the medium of A375 cells was measured by multiplexed ELISA 24 h after treatment with CDDP or MTX in the presence or absence of KIRA6 (1 μM). Data is expressed as log_2 (fold change) of MFI values.

In Western Blots β-actin was used as loading control. In all graphs values are presented as mean ± SD of at least n=3 independent biological replicates. Data is analyzed by one-way ANOVA followed by Dunnett's multiple comparison test in (A, E) and one-sample t-test in (D, F),$^{*}p<0.05$,$^{**}p<0.01$.

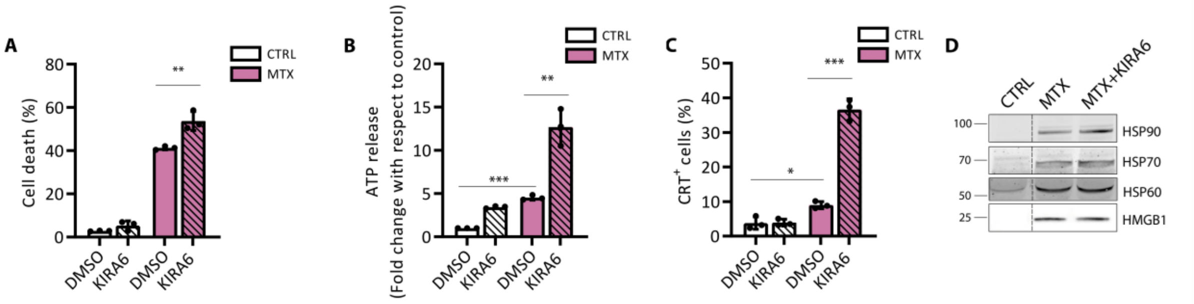

Supplementary Figure 5 (A) Cell death was assessed in CT26 cells 24 h after treatment with MTX in the presence or absence of KIRA6 (1 μM). **(B)** ATP secretion in the medium of CT26 was measured by luciferase-based assay at the indicated time points after treatment with MTX in the presence or absence of KIRA6 (1 μM). **(C)** CT26 cells treated with MTX in the presence or absence of KIRA6 (1 μM) were evaluated for the externalization of CRT in non-permeabilized cells by FACS staining 24 h after treatment. Dead cells were excluded from the analysis. **(D)** Representative Western Blot of danger signals released in the conditioned medium of CT26 cells 24 h after treatment with MTX in the presence or absence of KIRA6 (1 μM). Values are presented as mean ± SD of n=3 technical replicates**.** Data is analyzed by two-tailed Student's t-test, *p<0.05, **p<0.01,***p<0.001.

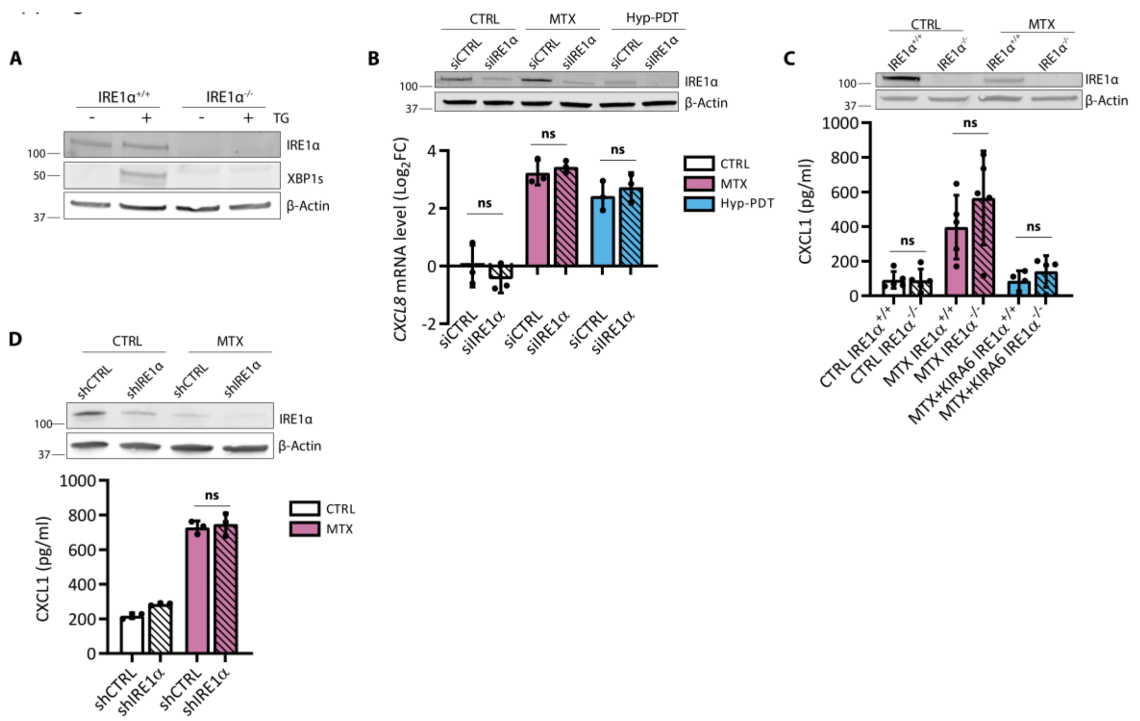

Supplementary Figure 6 (A) Representative Western Blot showing the ability of thapsigargin (TG, 2 μM) to promote the splicing of XBP1 (XBP1s) 8 h after treatment in IRE1α proficient and deficient A375 cells. β-actin was used as loading control. **(B)** The levels of *CXCL8* mRNA induction were assessed by qPCR in A375 transfected with scrambled siRNA (siCTRL) or with IRE1α targeting siRNA (siIRE1α) in basal condition or 4 h after treatment with MTX or Hyp-PDT. **(C)** CXCL1 secretion was measured by ELISA in conditioned medium of murine embryonic fibroblasts (MEFs) proficient or deficient for IRE1α 24 h after treatment with MTX (1 μM) in the presence or absence of IRE1α inhibitor KIRA6 (1 μM). **(D)** CXCL1 secretion was measured by ELISA 24 hr after treatment with MTX (5 μM) in conditioned medium of murine CT26 cells after short hairpin (sh)-RNA mediated silencing of IRE1α.

In all graphs values are presented as mean ± SD of at least n=3 independent biological replicates and analyzed by two-tailed Student's t-test, ns=not significant.

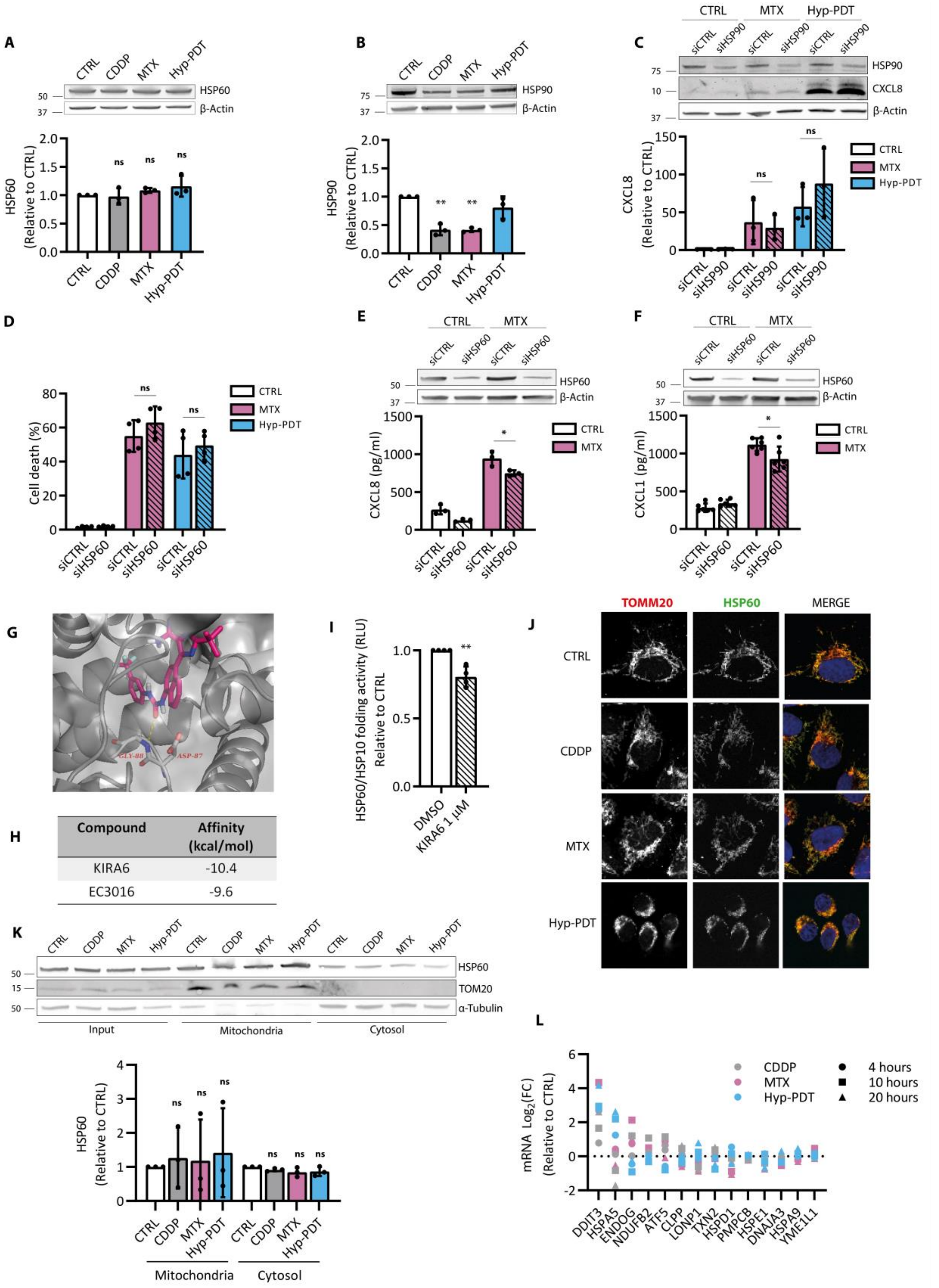

Supplementary Figure 7 (A-B) Representative Western Blot and quantification of intracellular HSP60 and HSP90 content in A375 cells in basal conditions and 24 h after treatment with CDDP, MTX, or Hyp-PDT. **(C)** Representative Western Blot and quantification of the impact of HSP90 targeted siRNA (siHSP90) with respect to scramble siRNA (siCTRL) on intracellular CXCL8 accumulation 4 h after treatment with MTX or Hyp-PDT.

Data is expressed as fold change over control incubated with siCTRL. **(D)** Cell death was assessed in A375 cells 24 h after treatment with MTX or Hyp-PDT upon transfection with scramble siCTRL or siHSP60. **(E)** CXCL8 secretion and **(F)** CXCL1 secretion were measured by ELISA in conditioned medium from HeLa and CT26, respectively, with siRNA mediated HSP60 knock-down 24 h after treatment with MTX. **(G)** Docking simulation shows hydrogen bond interaction between Gly88 of HSP60 and KIRA6 in yellow. **(H)** Binding energy of KIRA6 and EC3016 on HSP60 as predicted by docking simulation. **(I)** *In vitro* refolding activity of the HSP60/HSP10 chaperone complex after 1 h of incubation with heat-mediated unfolded substrate proteins in control condition or in the presence of KIRA6 (10 µM). Data is expressed as fold change compared to control. **(J)** Representative confocal microscopy images for the intracellular localization of HSP60 (Green) and TOMM20 (red) in A375 cells 4 h after treatment with CDDP, MTX, or Hyp-PDT. Nuclei were counterstained with DAPI (blue). Scale bar: 10 µm. **(K)** Levels of HSP60 in the cytosol and mitochondria in basal condition or 4 h after treatment with CDDP, MTX, or Hyp-PDT were assessed by Western Blot after subcellular fractionation. TOMM20 was used as mitochondrial marker and α-tubulin was used as cytosolic marker. **(L)** Gene expression obtained from RNAseq data representing the $\log_2$(fold changes) of gens relative to the induction of mitochondrial UPR upon treatment with CDDP, MTX, or Hyp-PDT compared to time-matched untreated control

In all graphs values are presented as mean ± SD of at least n=3 independent biological replicates. Data is analyzed by one-sample t-test in (A,B,I,K) and two-tailed Student's t-test in (C,D, E, F), *$p<0.05$, **$p<0.01$, ns= not significant.

Supplementary tables

Supplementary Table 1. Antibodies

Target	Species	Catalog Number	Company
CXCL8	Mouse	MAB-208	R&D systems
CXCL3	Rabbit	AV07037	Sigma-Aldrich
β-Actin	Mouse	A5441	Sigma-Aldrich
DR5	Rabbit	8074	Cell Signaling
cJUN	Rabbit	9165	Cell Signaling
IRE1	Rabbit	3294	Cell Signaling
pIRE1	Rabbit	NB100-2323	Novus Biologicals
XBP1s	Mouse	647501	Bioloegend
HSP60	Rabbit	15282-1-AP	Proteintech
HSP90	Mouse	ADI-SPA-830-F	Enzo Life sciences
TOMM20	Mouse	612278	BD Biosciences
HMGB1	Rabbit	ab18256	Abcam
pEIF2α	Rabbit	3597	Cell Signaling
EIF2α	Mouse	2103	Cell Signaling
ATF4	Mouse	97038	Cell Signaling
CHOP	Mouse	2895	Cell Signaling
cFOS	Rabbit	2250	Cell Signaling
α-Tubulin	Mouse	T6199	Sigma-Aldrich

IKK-β	Rabbit	8943	Abcam
Anti-rabbit AF647	Goat	A-21245	Thermofisher
Anti-mouse AF647	Goat	A-21235	Thermofisher
Anti-rabbit AF488	Goat	A-11008	Thermofisher
Anti-Rabbit IgG (H+L), DyLight 680 Conjugated	Goat	35571	Thermofisher
Anti-Mouse IgG (H+L), DyLight 800 conjugated	Goat	35521	Thermofisher
Anti-mouse IgG, HRP linked	Horse	7076	Cell Signaliing
Anti-rabbit IgG, HRP linked	Goat	7074	Cell Signaliing

Supplementary Table 2. PCR Primers

Gene	Forward (5' → 3')	Reverse (5' → 3')
CXCL2	CCATGGTTAAGAAAATCATCGAAA	TCCTTCCTTCTGGTCAGTTGGA
CXCL3	TCCCCCATGGTTCAGAAAATC	CTTCTTACTTCTCTCCTGTCAGTTGGT
CXCL8	GCAGAGGGTTGTGGAGAAGTTT	TTGGATACCACAGAGAATGAATTTTT
XBP1S	GGAGTTAAGACAGCGCTTGG	GTTCTGGAGGGGTGACAACT
18S	ATCCCTGAAAAGTTCCAGCA	CCCTCTTGGTGAGGTCAATG
